# A Digital Photo Activity Intervention for Nursing Home Residents With Dementia and Their Carers: Mixed Methods Process Evaluation

**DOI:** 10.2196/56586

**Published:** 2025-04-16

**Authors:** Josephine Rose Orejana Tan, David P Neal, Maria Vilmen, Petra Boersma, Teake P Ettema, Robbert J J Gobbens, Sietske A M Sikkes, Rose-Marie Dröes

**Affiliations:** 1 Department of Psychiatry Amsterdam Public Health Research Institute Amsterdam UMC, location Vrije Universiteit Amsterdam The Netherlands; 2 eHealth Living & Learning Lab Department of Medical Informatics Amsterdam UMC Amsterdam The Netherlands; 3 Lectorate Health and Well-being of Frail Elderly People Faculty of Health, Sports and Social Work Inholland University of Applied Sciences Amsterdam The Netherlands; 4 Program Learning, Improvement & Implementation Ben Sajet Centrum Amsterdam The Netherlands; 5 Zonnehuisgroep Amstelland Amstelveen The Netherlands; 6 Department of Family Medicine and Population Health Faculty of Medicine and Health Sciences University of Antwerp Antwerp Belgium; 7 Tranzo Tilburg University Tilburg The Netherlands; 8 Department of Clinical, Neuro- & Developmental Psychology Faculty of Behavioural and Movement Sciences Vrije Universiteit Amsterdam Amsterdam The Netherlands; 9 Neurology Alzheimer Center Amsterdam Vrije Universiteit Amsterdam, Amsterdam UMC Amsterdam The Netherlands; 10 Amsterdam Neuroscience, Neurodegeneration Amsterdam The Netherlands

**Keywords:** dementia, psychosocial interventions, nursing home, process evaluation, social interaction, photos, art

## Abstract

**Background:**

Within the framework of a randomized controlled trial investigating the impact of a digital, psychosocial photo activity intervention for residents living with dementia in nursing homes and their informal and formal carers, a process evaluation was conducted to determine factors that affected the implementation of the intervention and potentially influenced the intervention outcomes.

**Objective:**

By tracing facilitators and barriers to implementation, the study also aimed to inform future implementation of the photo activity intervention.

**Methods:**

Following Medical Research Council guidance, mixed methods were used to investigate context, implementation, and mechanism-of-impact factors during the photo activity intervention via the Fotoscope web application versus a general conversation activity (control). Google Analytics was set up to gain insight into how the Fotoscope web application was used in practice. For quantitative data, descriptive statistics were calculated and differences between groups tested. For qualitative data, thematic analysis was performed.

**Results:**

In total, 163 semistructured interviews were conducted with residents (photo activity group: n=29, 17.8%; control: n=29, 17.8%), formal carers (photo activity group: n=23, 14.1%; control: n=27, 16.6%), and informal carers (photo activity group: n=28, 17.2%; control: n=27, 16.6%). Regarding contextual factors, a minority of formal carers in both groups (photo activity group: 4/18, 22%; control: 9/24, 38%) mentioned time and workload as barriers to implementing the intervention. Regarding implementation, 86% (25/29) of the residents in the intervention group felt that the digital photo activity worked well on a tablet. Informal carers from both groups wanted more intervention updates from formal carers. The majority of formal carers from both groups were satisfied with how the training and activities were implemented. Regarding the mechanisms of impact, residents in the photo activity group (27/29, 93%) felt significantly more positive about the conversations with their carer (*U*=533.0, *z*=2.865, *r*=0.39; *P*=.004). Formal carers in the photo activity group (20/23, 87%) got to know the resident better (*U*=390.5, *z*=2.114, *r*=0.302; *P*=.04) compared to the formal carers in the control group (21/27, 78%). Formal carers in the photo activity group (23/50, 46%) gave a significantly higher rating to the digital photo activity as a way of getting to know the resident living with dementia better (median 9.00, IQR 7-9; *U*=419.0, *z*=2.169, *r*=0.307; *P*=.03) compared to formal carers in the control group (27/50, 54%; median 8.00, IQR 6-8). Finally, the majority of formal carers in the photo activity group (14/18, 78%) agreed that the Fotoscope app can be used as part of care activities in the nursing home.

**Conclusions:**

The work invested by formal carers in implementing the photo activity did not seem to differ greatly compared to implementing a general conversation activity, suggesting that the digital photo activity, as an easy-to-implement and enjoyable intervention, could be widely implemented and disseminated in nursing homes.

**International Registered Report Identifier (IRRID):**

RR2-https://doi.org/10.1186/s12877-021-02632-w

## Introduction

### Background

The photo activity is a psychosocial intervention based on an earlier project by a visual artist (known as *Photographic Treatment* [1]) for nursing home residents with dementia and their informal and formal carers. It is designed to facilitate social interaction [2]. In a small-scale pilot study (n=20), nursing home residents viewed printed, artistic, generic photos related to their personal interests [2]. The study found that when residents with dementia viewed person-centered generic photos (ie, not personal or family photos but images of subjects related to personal interests) as opposed to non–person-centered photos during conversations with their informal or formal carers, they tended to exhibit more positive effects on social interaction, mood, speech, and negative behavior [2]. In recent decades, psychosocial interventions have been influenced in part by the many technological developments aimed at improving the social health and quality of life of people with dementia [3-6]. Examples include the use of social robots [7], exergaming [8], and online art gallery activities [9]. However, few studies have looked into the effects of psychosocial interventions using technology for nursing home residents with dementia [10-12]. The photo activity is an example of a psychosocial intervention offered in nursing homes that was recently digitally adapted through the development of the Fotoscope app [13].

The development of the Fotoscope app allows for upscaling and wider dissemination of the digital photo activity intervention (ie, photo activity using digital photos, rather than printed photos). Using the app, informal and formal carers can easily conduct the digital photo activity with residents by accessing a database of artistic, black-and-white, generic photos and making a person-centered selection of digital photos to view with the residents [13], as opposed to having a limited collection of printed photos. However, before disseminating the digital photo activity intervention, it is important to evaluate its effectiveness and to consider facilitators and barriers to implementing it in daily nursing home care.

It is argued that alongside evaluating an intervention’s effects, process evaluations are crucial to look at how and under what conditions an intervention was implemented, to make the outcomes reproducible, and to learn about factors that facilitated or impeded the implementation of the intervention, all of which will inform the interpretation of trial outcomes and future upscaling [14-16]. The importance of conducting process evaluations alongside randomized controlled trials (RCTs) was emphasized in the revised UK Medical Research Council (MRC) guidance for process evaluations of complex interventions [17]. In addition to tracing facilitators and barriers to implementation, process evaluations allow for the identification of contextual factors that may have influenced the implementation of the intervention and its outcomes, as well as the mechanisms underlying the intervention’s impact [16,17]. *Implementation* refers to the ways and processes in which the intervention was delivered in practice, how frequently it was delivered, and whether it was delivered as intended. *Mechanisms of impact* refers to the aspects of the intervention that created change in practice or in the target population, investigating which would help in understanding future replications of the intervention. Finally, *context* refers to factors outside of the intervention that may facilitate or hinder the implementation [17].

As technology for dementia care continues to advance, along with RCTs evaluating the effectiveness of care technology [3,10], it is becoming more common to include process evaluations alongside such trials; for example, process evaluations based on the MRC guidance have been conducted in studies evaluating the use of different kinds of technology in psychosocial care for people with dementia in the community or long-term care settings [18-21].

### Objectives

This paper reports on a process evaluation based on the MRC guidance [17], which was carried out alongside a pilot RCT (Netherlands Trial Register: NL9219 [22]) investigating the feasibility and impact of the digital photo activity intervention. The implementation of the photo activity intervention in comparison with the implementation of a control intervention (a general conversation activity without photos) was evaluated. The purpose of this study was to identify factors that either facilitated or hindered the implementation of the photo activity intervention compared to a general conversation activity, including contextual, implementation, and mechanism-of-impact factors, to generate insights to interpret RCT outcomes and to inform further implementation and dissemination of the intervention.

The study aimed to answer the following research questions (RQs):

- RQ1: How did factors external to the interventions affect the implementation of the interventions (context)?
- RQ2: How did residents and their informal and formal carers assess the preparation, introduction, and delivery of the interventions? (implementation)?
- RQ3: How did the residents with dementia and their informal and formal carers experience and value the digital photo activity versus the general conversation activity (mechanisms of impact)?

## Methods

### Design of the Process Evaluation

**Figure 1:**
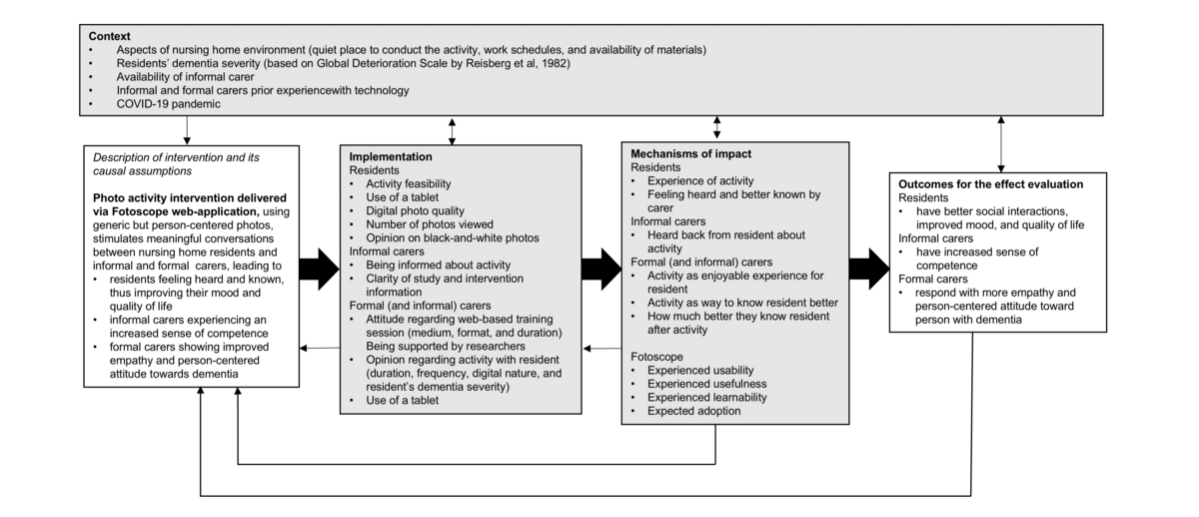
Contextual, implementation, and mechanism-of-impact factors, based on the Medical Research Council guidance for process evaluation, that could facilitate or hinder the implementation of the digital photo activity in the nursing home.

Following MRC guidance [17] (Figure 1 [23]), we used a mixed methods approach to investigate contextual, implementation, and mechanism-of-impact factors because the combination of quantitative and qualitative data would allow us to gain a more comprehensive understanding of the way the photo activity, as a complex intervention, is organized, offered, and implemented in the clinical setting [17,24]. Using mixed methods can also provide the flexibility needed in working with nursing home residents with dementia, ensuring that they are able to contribute their insights regarding the intervention [24,25]. The reporting and writing of this study was guided by the Good Reporting of a Mixed Methods Study (GRAMMS) checklist [26].

During the 4-week intervention period, analytics data were collected on Fotoscope app use and interactions of carer-resident pairs assigned to the photo activity intervention. After the intervention, qualitative and quantitative data were collected through semistructured interviews. The full protocol for the RCT, including the process evaluation, has been published elsewhere [13].

### Ethical Considerations

This research was approved by the Vrije University Medical Center’s Ethics Review Committee (2020.221) and embedded within the Amsterdam Public Health Research Institute (SQC2020-039). All participants provided informed consent. For individuals with dementia who lacked the capacity to consent, their legal representative signed the consent form on their behalf.

For privacy and confidentiality, data are anonymized, coded, and stored securely. Financial compensation was given to participating nursing home wards for their time (€100 [US $108.7] per participating resident with dementia who completed an intervention round).

### Participants

The study aimed to recruit nursing home residents in the Netherlands with dementia at severity levels 4, 5, or 6 on the Global Deterioration Scale (GDS) [23], along with their informal and formal carers. To do this, the researchers worked with interested regional and national nursing home organizations. The study was presented to individual nursing home wards, where ward managers, in consultation with their teams, determined their willingness and capacity to participate. In total, 20 nursing home wards from 3 large care organizations took part in the study. They were asked to recruit formal carers to fulfil the following roles: coordinator (served as the first point of contact, recruited informal carers and residents with dementia for the study by providing written and oral information about the study, and assessed the dementia severity of participating residents), intervention providers (2 formal carers randomized to deliver either the photo activity or control activity), and independent assessor (a formal carer not involved in delivering either activity, who conducted blind assessments of residents’ daily functioning in the ward for the pilot RCT by filling out web-based questionnaires). Informal carers of residents at the participating care organizations were also allowed to provide either the photo activity or control activity if they wished. Researchers contacted residents and informal carers about their participation in the study only after the coordinator obtained their informed consent. The photo activity and control activity were provided by formal carers (or informal carers if they wished to provide the interventions themselves) to the residents twice a week for 4 weeks. Residents as well as informal and formal carers who provided either activity and completed the 4-week intervention period were then recruited for the process evaluation by the researchers and were invited to complete the semistructured interviews via online video calls or telephone calls. The formal carers who participated as independent assessors were only involved in the pilot RCT and not in the process evaluation.

Pairs of residents with the same dementia severity who had lived in the nursing home for at least 1 month were recruited by the coordinator. After providing informed consent, they were randomly assigned to either the experimental or control condition through a lot-drawing process. The only exclusion criteria were severe vision or hearing problems. Informal carers were assigned to the same condition as the resident.

The sample size for the RCT was determined based on the results of the photo activity pilot study. Large positive effect sizes were found for the INTERACT subscales social interaction (Cohen *d*=0.86) and reduced negative behavior (Cohen *d*=0.85) [2]. On the basis of these effect sizes and an expected dropout rate of 10% over 6 weeks, a power calculation (power=0.80; α=.05; Cohen *d*=0.80) indicated that 45 residents per group (experimental and control) were needed. All residents and their carers involved in the RCT were invited to participate in the process evaluation.

### Interventions

#### Photo Activity (Experimental Intervention) and the Fotoscope Web Application

**Figure 2:**
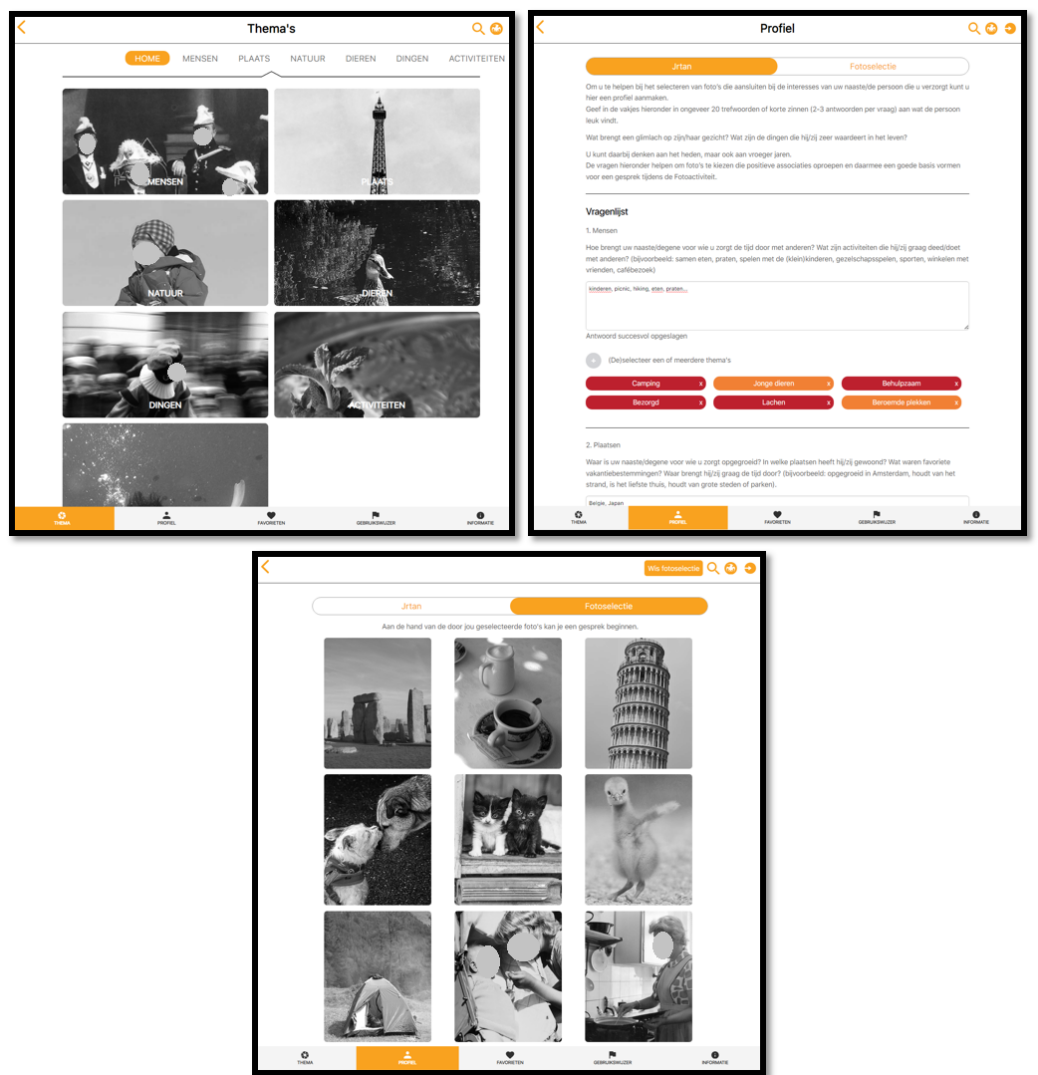
Screenshots of the Fotoscope web application’s Themes, Profile, and Photo Selection pages. Photos in the Fotoscope web-application were publicly available on the internet and collected by the artist, or were taken by the artist herself. No personal photos of participants were used.

The photo activity intervention engaged residents and their carers in a 1-on-1 conversation about artistic, person-centered generic photos in the Fotoscope web application. For the digital photo activity, the Fotoscope database was expanded to 1500 black-and-white photos, which were organized according to 7 main themes (people, places, nature, animals, activities, things, and experiences). The Fotoscope web application features multiple sections, such as Themes, Profile, Favorites, User Guide, and Information (Figure 2). The Themes page displays the 7 main photo categories and subcategories. The Profile page contains questions designed to learn more about the residents’ personal interests and features a selection tool that allows carers to easily select photos that match the residents’ interests and save them in the Photo Selection tab. The Favorites page displays photos that elicit positive reactions from the resident, which the carer can add during or after their conversation. The User Guide provides instructions on using the Fotoscope web application and offers tips on person-centered communication. The Information page contains details about copyright and the researchers’ contact information. Additional features include a search function (carers can enter keywords to find a photo) and a “joker” function (a collection of photos that have been found to elicit positive reactions).

#### General Conversation Activity (Control Intervention)

The control intervention also involved residents and their carers in a 1-on-1 conversation but without the Fotoscope web application and generic photos. The carer was encouraged to have an open conversation with the resident based on general topics and to allow the resident to lead the conversation.

### Data Collection Methods

Data for the process evaluation were collected via a demographic questionnaire, semistructured interviews, Google Analytics, and a carer self-report registration form (Multimedia Appendix 1).

#### Demographic Questionnaire

Questions for the demographic questionnaire administered at baseline (T0) were adapted from The Older Persons and Informal Caregivers Survey Minimum Dataset [27], with additional questions pertaining to relevant medical information for the residents with dementia. For residents, information on sex, age, education, years in the nursing home, type of dementia, and severity of dementia was collected. For informal carers, information on sex, age, education, relationship to residents, and employment status (whether they had paid work) was collected. For formal carers, information on sex, age, education, work function, and years of experience in psychogeriatrics was collected.

#### Semistructured Interview

The questions for the semistructured interview, which was conducted after the 4-week intervention period (T1), were adapted from existing interview guides used in a previous process evaluation study on a digital psychosocial intervention for people with dementia and their caregivers and pilot-tested [18]. The questions were adapted by JROT, RMD, and PB to fit the photo activity trial. On the basis of the MRC guidance for process evaluation [17], context, implementation, and mechanism-of-impact factors (including usability, usefulness, learnability, and adoption of the Fotoscope app) were explored through a mix of open-ended and closed-ended questions (Multimedia Appendix 2). An example of a closed-ended question for a resident with dementia who participated in the photo activity intervention is “Do you think your carer got to know you better by having a regular conversation with you about the photos together?”; the corresponding closed-ended question for a resident with dementia in the control condition was “Do you think your carer got to know you better by having regular conversations with you on general topics?” The answer options for these questions were “A lot better,” “Better,” “A little better,” and “No, not better.”

#### Google Analytics

The Fotoscope web application was linked to a Google Analytics account to measure the carers’ use of, and interactions with, the app during the trial. To protect residents’ privacy, IP addresses were masked, and codes rather than names were used to log in. To gain insight into carers’ engagement with the Fotoscope web application (*implementation*), data on the number of sessions per user and average session durations were collected. In addition, dates of each session, as well as session start times, were recorded for each user.

To determine whether carers used the Fotoscope web application to get to know residents better (*mechanisms of impact*), unique page views of the Profile and Favorites pages were tracked in Google Analytics. To determine whether carers found the pages and functions of the Fotoscope web application useful (*usefulness*), the number of unique views of these pages was recorded as an indication. To determine whether carers needed additional guidance on using the Fotoscope web application, the number of unique page views of the User Guide and Information page was recorded (*learnability*). Finally, information on device type was collected (*context*) to know whether the Fotoscope web application was used by carers on a tablet, as advised.

#### Carer Self-Report Registration Form

Carers were asked to record the dates, times, and duration of the activities with the residents on a standard record form to track the time spent on the activities and the number of sessions conducted (*implementation*)*.* If a session was missed, the intervention discontinued, or the resident dropped out, they were asked to document the reason on the form.

### Procedure

The pilot RCT in February 2021 was disrupted by the COVID-19 pandemic. In the Netherlands, nursing homes were placed under quarantine, restricting visitor access [28]. The trial protocol was adapted to an online format. Carers assigned to the photo activity attended a 1.5-hour web-based training session where they were introduced to the photo activity intervention and procedure [2] and were shown an online demonstration of the Fotoscope web application. General communication tips were discussed, followed by more specific person-oriented communication skills for interacting with people with dementia [29]. Carers were then given personal log-in credentials and passwords for the Fotoscope web application—1 set for their own use to enable them to become familiar with the app and another for each resident participating in the photo activity. Each resident’s Fotoscope user account was linked to a unique user ID in Google Analytics, and only the researchers had access to the key linking the IDs to specific residents. Carers in the photo activity group were instructed to prepare for the first session by completing the Fotoscope Profile page with input from family members and selecting a minimum of 15 photos based on the residents’ interests. Formal carers in the photo activity group were instructed to update the informal carers (by telephone or when they visited the resident) on how the residents responded to the activity, at least once during the 4-week intervention.

The carers assigned to the control activity attended a 1-hour online training session where the researchers explained the procedure for the general conversation activity and provided general communication tips.

In both conditions, carers were advised to conduct the activities with the resident twice a week for 4 weeks, with each session lasting 30 minutes. They were also advised to invite the resident to a quiet room in the ward where they could conduct the activity without disruption. Carers assessed residents’ feelings before and after each session using the Smiley Face Assessment Scale (SFAS) [30]. Trained female and male research assistants observed the first and last sessions via video calls, assessing social interaction between the resident and carer during the activity using the INTERACT observation scale [13]. After these sessions, carers in the photo activity group received feedback from the research assistants on how to improve person-centered communication based on their observations. At the end of the final session, the residents with dementia were interviewed during the video call for 5 to 10 minutes by the research assistant, using questions from the semistructured interview guide. Both informal and formal carers either completed the digital form or participated in a 20-minute interview via telephone or video call at a time convenient to them within a week after the final intervention session. Carers who were unable to attend an interview were asked to submit a written narrative account of their experience in response to the questions from the interview guide. Formal carers who conducted either the photo activity or control activity with >1 resident were asked to complete a separate interview for each resident because some of the questions could be answered differently depending on the resident.

The independent assessors completed questionnaires on the residents’ daily functioning in the ward at baseline (T0), after the intervention (T1), and at the 2-week follow-up (T2). Results relevant to the observations of the independent assessors will be reported in a separate paper on the effect evaluation of the digital photo activity.

### Analysis

#### Quantitative Analyses

Participants’ background characteristics were analyzed using descriptive statistics in SPSS software (version 28.0; IBM Corp). Differences between the photo activity and control activity conditions were tested using 2-tailed *t* tests for continuous variables, Mann-Whitney *U* tests for ordinal variables, and Pearson chi-square tests for nominal variables. Effect sizes were interpreted using Cohen *d* for *t* tests (small=0.2, medium=0.5, and large is ≥0.8), *r* for Mann-Whitney *U* tests (small=0.1, medium=0.3, and large is ≥0.5), and phi coefficient (φ; for 2 × 2 tables) or Cramér V (for tables larger than 2 × 2) for Pearson chi-square tests, with the effect size based on the number of categories involved.

Data from closed-ended questions were summarized as percentages or as means and SDs, depending on the measurement level. As not all questions were comparable between the photo activity and control groups—some questions were intervention related—only selected comparable closed-ended questions were analyzed for between-group differences for each of the participant groups (residents, formal carers, and informal carers) using chi-square tests, *t* tests, or Mann-Whitney *U*-tests.

Google Analytics recorded information from users identified as “carer-resident” pairs with unique user IDs. The first sessions for the trial started on February 15, 2021; however, because of a delay in setting up the live environment for Google Analytics for the Fotoscope web application, data collection only started on March 12, 2021. Data collection was completed on August 6, 2023. For privacy reasons, Google Analytics only retains data for 26 months [31], and data were not backed up. As a result, data from 10 users who participated in the trial from March to October 2021 were automatically deleted before analyses were conducted in August-September 2023.

Observed session durations extracted from Google Analytics were matched to the dates and times recorded in the carer self-report registration forms. The agreement between total observed and self-reported durations (in minutes) was analyzed using the Spearman rho correlation coefficient. Percentages of total unique page views for the main pages were calculated from the raw data. The frequency of selecting main photo categories was also calculated from raw data (unique page views) and summarized as percentages. Individual reports were viewed to track events or actions taken by specific users during each session (eg, viewing the Profile page or opening a photo from the Photo Selection page). These actions were recorded per user on a binary (yes or no) scale and reported as the percentage of sessions in which events of interest (eg, adding a photo to the Favorites or Photo Selection pages) occurred. The use of a tablet or a different device for the Fotoscope web application was counted per user and summarized as percentages.

#### Qualitative Analyses

Data from the open-ended questions in the semistructured interviews were analyzed using the framework of theoretical thematic analysis, which was defined by Braun and Clarke [32] as thematic analysis that is built upon existing theoretical work in the literature. The researchers (JROT: PhD student, English speaking; MV: master’s student, English and Dutch speaking; and SAMS: senior researcher, English and Dutch speaking) worked together following the 6 phases of thematic analysis [32]. The researchers individually familiarized themselves with the data (phase 1) by reading all answers to the open-ended and closed-ended questions of the semistructured interviews. Next, they generated initial codes (phase 2) based on aspects of the MRC guidance (contextual, implementation, and mechanism-of-impact factors) [17], which served as the guiding framework for this thematic analysis. The researchers studied the data to examine how the data fit within the MRC guidance framework; thus searching for themes (phase 3) involved assessing whether the data from the semistructured interviews fit within the main themes of context, implementation, and mechanisms of impact factors.

Themes and codes were reviewed (phase 4) by the 3 researchers through several online meetings. Themes and subthemes were then named and defined (phase 5). As stated by Braun and Clarke [32], these phases are recursive, and the researchers moved back and forth between phases as necessary. Reporting of the results (phase 6) followed the COREQ (Consolidated Criteria for Reporting Qualitative Research) guidelines [33]. MV and SAMS worked with the original data in Dutch, while JROT used an English translation generated using DeepL [34]. The researchers agreed on the English translations of the quotes and developed the codes and themes in English.

## Results

### Characteristics of Participants

**Figure 3:**
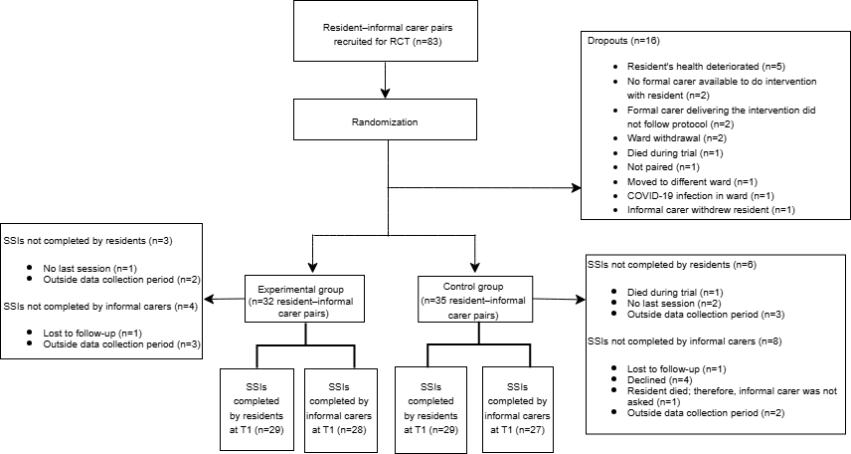
Recruitment of residents and informal carers, showing randomization into the experimental and control groups, as well as dropouts and reasons for dropping out. RCT: randomized controlled trial; SSI: semistructured interview; T1: after the intervention.

Of the 83 resident–informal carer pairs recruited for the RCT, 16 (19%) dropped out for different reasons after randomization (Figure 3). Among the experimental group (n=32 resident–informal carer pairs), 29 residents and 28 informal carers completed semistructured interviews after the final session. Among the control group (n=35 resident–informal carer pairs), 29 residents and 27 informal carers completed the interviews. Reasons for not completing the interviews are provided in Figure 3. No informal carers delivered the experimental or control intervention, partly due to restrictions in visiting the nursing home during the COVID-19 pandemic and partly because of other reasons, such as not having enough time to visit the ward as needed because of their job or other concurrent tasks.

**Figure 4:**
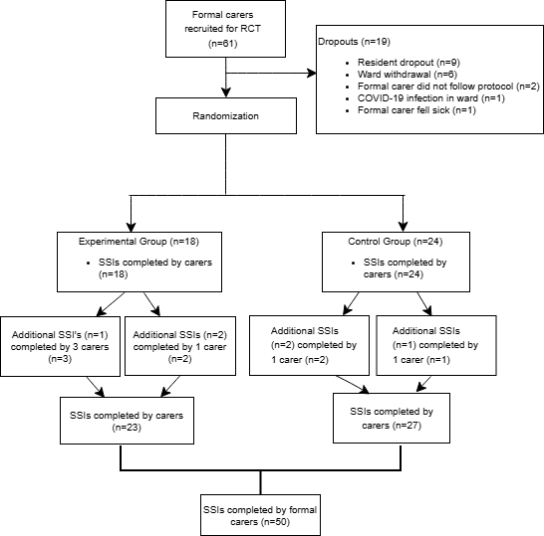
Recruitment of formal carers, showing randomization into the experimental and control groups, as well as dropouts and reasons for dropping out. RCT: randomized controlled trial; SSI: semistructured interview.

Of the 61 formal carers recruited for the RCT, 42 (69%) completed the trial and the semistructured interviews (carers experimental group: n=18, 43%; carers control group: n=24, 57%). As a number of carers conducted either the experimental activity or control activity with >1 resident, they were asked to complete a separate interview for each resident; hence, 50 semistructured interviews with residents (experimental group: n=23, 46%; control group: n=27, 54%; Figure 4) were completed by formal carers.

Tables 1, 2 and 3 present the background characteristics of the residents, informal carers, and formal carers, respectively, who completed the semistructured interviews. There were no significant differences between the experimental and control groups.

**Figure 5:**
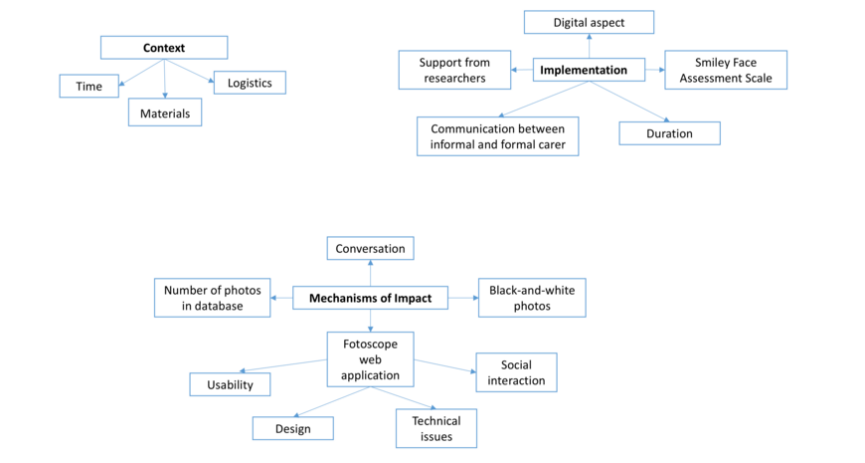
Thematic map from qualitative analyses showing the main themes (context, implementation, and mechanisms of impact), subthemes, and subsubthemes.

Figure 5 shows the thematic map with the 3 main themes identified in the qualitative thematic analysis, while Multimedia Appendix 3 provides a detailed overview of the themes, subthemes, and sample data extracts. Relevant qualitative results from the thematic analysis are integrated with the quantitative results in the following subsections.

**Table 1:** Background characteristics of nursing home residents in both the experimental and control groups who completed the semistructured interviews after the 4-week intervention.

Characteristics			Experimental group (n=29)		Control group (n=29)		Difference tests													*P* value^a^	
							Chi-square (*df*)			*t* test (*df*)			*U*			*z*					
Sex: female, n (%)			24 (83)		22 (76)		0.1^b^ (1)			—^c^			—			—			.75		
Age (y), mean (SD; range)			84.03 (7.36; 68-96)		84.38 (7.48; 62-96)		—			−0.177 (56)			—			—			.86		
**Education, n (%)**								—			—			308.0			−1.502				.13
	Elementary	7 (24)		6 (21)																	
	Lower secondary education	19 (66)		11 (38)																	
	Higher secondary education	1 (3)		5 (17)																	
	University	2 (7)		5 (17)																	
	Missing	0 (0)		2 (7)																	
Time spent in nursing home (y), mean (SD; range)			1.714 (1.460; 0.3-7.0)		2.038 (2.090; 0.2-10.0)		—			—			365.5			−0.427			.68		
**Type of dementia, n (%)**								1.1^b^ (1)			—			—			—				.30
	Alzheimer disease	17 (59)		21 (72)																	
	Other	12 (41)		7 (24)																	
	Missing	0 (0)		1 (3)																	
**Severity of dementia (GDS^d^ scores), n (%)**								—			—			398.5			−0.380				.70
	4	4 (14)		3 (10)																	
	5	15 (52)		15 (52)																	
	6	10 (34)		11 (38)																	

^a^Significance level set at *P*<.05.

^b^Chi-square test with Yates continuity correction.

^c^Not applicable.

^d^GDS: Global Deterioration Scale.

**Table 2:** Background characteristics of informal carers in both the experimental and control groups who completed the semistructured interviews after the 4-week intervention.

Characteristics		Experimental group (n=28)	Control group (n=27)	Difference tests									*P* value^a^
				Chi-square (*df*)		*t* test (*df*)		*U*		*z*			
Sex: female, n (%)		21 (75)	20 (74)	0.0^b^ (1)		—^c^		—		—		.99	
Age (y), mean (SD; range)		57.04 (9.03; 31-74)	60.74 (11.4; 27-84)	—		−1.341 (53)		—		—		.39	
**Education, n (%)**					—		—		317.5		−1.108		.22
	Elementary	0 (0)	2 (7)										
	Lower secondary education	17 (61)	9 (33)										
	Higher secondary education	3 (11)	4 (15)										
	University	8 (29)	12 (44)										
	Missing	0 (0)	0 (0)										
**Relationship to resident, n (%)**					1.6 (3)		—		—		—		.65
	Husband, wife, or partner	4 (14)	6 (22)										
	Son or daughter	3 (11)	4 (15)										
	Daughter-in-law or son-in-law	18 (64)	16 (59)										
	Other	3 (11)	1 (4)										
Paid work: yes, n (%)		21 (75)	15 (56)	1.5^a^ (1)		—		—		—		.22	

^a^Significance level set at *P*<.05.

^b^Chi-square test with Yates continuity correction.

^c^Not applicable.

**Table 3:** Background characteristics of formal carers in both the experimental and control groups who completed the semistructured interviews after the 4-week intervention.

Characteristics		Experimental group (n=18)	Control group (n=24)	Difference tests									*P* value^a^
				Chi-square (*df*)		*t* test (*df*)		*U*		*z*			
Sex: female, n (%)		17 (94)	23 (96)	0.0^b^ (1)		—^c^		—		—		.99	
Age (y), mean (SD; range)		45.7 (13.0; 20-62)	43.6 (15.1; 18-63)	—		−0.470 (40)		—		—		.64	
**Education, n (%)**					—		—		194.5		−0.843		.40
	Elementary	0 (0)	0 (0)										
	Lower secondary education	16 (89)	19 (79)										
	Higher secondary education	1 (6)	2 (8)										
	University	1 (6)	3 (12)										
	Missing	0 (0)	0 (0)										
**Job role, n (%)**					2.5 (3)		—		—		—		.48
	Carer	6 (33)	5 (21)										
	Nurse	1 (6)	0 (0)										
	Activity supervisor	3 (17)	6 (25)										
	Other	8 (44)	13 (54)										
Experience (y) in psychogeriatrics, mean (SD; range)		5.22 (4.62; 1.0-16.0)	10.75 (11.13; 1.0-40.0)	—		—		165.5		−1.297		.20	

^a^Significance level set at *P*<.05.

^b^Chi-square test with Yates continuity correction.

^c^Not applicable.

### Results From Analysis of Semistructured Interviews and Google Analytics Data

#### Overview

Multimedia Appendix 2 presents all answers to the closed-ended questions from the semistructured interviews summarized as percentages or as means and SDs.

In total, 41 users (representing carer-resident pairs) were given a Fotoscope account. Of these 41 users, 38 (93%) were recorded as accessing the app (of the remaining 3 users, 2, 67% had started the intervention before Google Analytics was set up; and 1, 33% included a resident who dropped out before starting the intervention). As data of 10 (26%) of the 38 users were no longer available at the time of evaluation, we analyzed the data of 28 (74%) users. Of these 28 users, 3 (11%) had partial or missing data. Of the remaining 25 user pairs, 4 (16%) had residents who dropped out (n=2, 50% dropped out before the activities started due to worsening conditions; n=1, 25% dropped out after 3 sessions due to worsening conditions; and n=1, 25% dropped out after 3 sessions because the formal carer was too busy); therefore, they were excluded from analysis, leaving data from 21 (84%) users (representing carer-resident pairs) for the final analysis.

#### Contextual Factors

The majority of the residents (experimental group: 23/29, 79%; control group: 27/29, 93%) found the space for viewing photos with their formal carers or having a conversation with them to be comfortable and quiet.

Most of the informal carers in the photo activity group (13/28, 46%) experienced no barriers to talking to the formal carers about the residents’ personal interests or their experience with the photo activity, while some cited barriers, including a lack of time (7/28, 25%) and “other reasons” (4/28, 14%), or did not respond (4/28, 14%). The majority of informal carers in the control group (15/27, 56%) reported no barriers to talking to the formal carers about the residents’ experience with the general conversation activity, while those who responded with “other reasons” (10/27, 37%) reported not knowing that it was an option to ask the formal carers about the general conversation activity with the residents. Other reported barriers included being on vacation (1/27, 4%) and a lack of time (1/27, 4%).

The majority of formal carers in the photo activity group (10/18, 56%) reported using a tablet “a lot” previously. The remaining responded as follows: “a little” (5/18, 28%), “hardly” (1/18, 6%), or “not at all” (2/18, 11%). The majority of the formal carers in both the photo activity group (14/18, 78%) and the control group (15/24, 63%) reported experiencing no barriers in conducting the activities. In the thematic analysis, formal carers from both groups who reported experiencing barriers mentioned difficulty in planning time for the activities, given their busy schedules in the ward, sometimes even having to find the time outside of their shifts to conduct the activity with the resident:

Planning the photo activity is very important because of our workload. Time and sufficient staff presence are very essential to consider.

Formal carer 109

Finding a quiet place to conduct the activity seemed to be more difficult for formal carers in the control group, while formal carers in the photo activity group mentioned difficulty in arranging materials for the activity, such as finding an available tablet in the ward (we had offered to lend tablets to the wards at the start of the study, but none chose this option).

#### Implementation Factors

The majority of the residents (25/29, 86%) reported that the photo activity went well as an activity on the tablet, and the majority (21/29, 72%) also reported seeing the photos clearly and finding them sufficiently large and sharp. Moreover, the majority (23/29, 79%) reported viewing a “good” amount of photos with their carers, and most (14/29, 48%) felt “neutral” about the black-and-white photos.

The majority of the informal carers (experimental group: 25/28, 89%; control group: 20/27, 74%) felt well informed about the activities that the residents performed in the nursing home during the intervention period. However, the thematic analysis revealed that informal carers in both groups desired more communication and updates from the formal carers regarding the residents’ engagement in the activities.

A majority of formal carers in both the photo activity (12/18, 67%) and control (20/24, 83%) groups felt that the medium and format of the training sessions were effective (Table 4). More than half of the formal carers in the photo activity group were positive about the online training sessions and rated them as “good” (5/18, 28%) or “satisfactory” (8/18, 44%), and the majority (12/18, 67%) also felt that the training duration was “just right.” A large majority of the formal carers in the control group also rated their online training sessions positively (824, 33% rated it as “good”; and 14/24, 58% rated it as “satisfactory”), and a large majority (20/24, 83%) also felt that the training duration was “just right.” Formal carers from both groups expressed a preference for a live training session and in-person support or demonstration. A majority of the formal carers from both the photo activity (14/18, 78%) and control (16/24, 67%) groups felt that the support provided by the researchers and resources was satisfactory. In the thematic analysis, several formal carers in both the photo activity and control groups commented on the duration of the online training sessions and the duration of the activity itself. Regarding the duration of the online training sessions, they seemed to agree that it should be shorter for both groups. The comments for activity duration were mixed. Formal carers in both the photo activity and control groups appreciated the support provided by the researchers and resources. A formal carer in the control group suggested incorporating a video example in the training sessions to demonstrate how the general conversation activity could be conducted, while a formal carer in the photo activity group suggested expanding the manual to include instructions on how to download the Fotoscope app on a new device.

The majority of the formal carers (16/23, 70%) found that the photo activity, using the Fotoscope app, was “easy to implement.” While the carers were positive about the app, a theme revealed by the thematic analysis was that they felt that the Fotoscope app was “not for everyone”:

Very good [app]. Albeit not suitable for everyone. Everything digital, this is something that the target group is not familiar with.

Formal carer 5

Formal carers who expressed this concern had conducted the photo activity with residents who had a GDS score of 5 or 6 [23]. A minority of carers in both groups also reported that residents with a GDS score of 5 or 6 had a hard time understanding the SFAS [30] and found it difficult to choose a face that represented how they felt in the moment. Thematic analysis revealed that more formal carers from the control group reported that it was difficult for the residents to understand the semistructured interview questions. Formal carers who conducted the activities with multiple residents also reported that completing the interview multiple times was a burden.

Table 4 presents results from the statistical analysis of differences between the formal carers in the photo activity group and those in the control group in terms of how they felt about the medium and format of the training sessions, the activity duration, the 4-week activity period, and the activity frequency. No significant differences were found (Table 4).

The relationship between the total duration of self-reported app use as measured by the carer self-report registration form and the corresponding (ie, matched to the date and time recorded in the self-report form) total duration of observed app use as measured by Google Analytics was investigated using the Spearman rho correlation coefficient. There was no significant correlation between the 2 variables (n=21; *r*=−0.108; *P*=.64).

The Google Analytics data showed that desktop computers were used most of the time to access the Fotoscope app (19/32, 59%), followed by tablets (8/32, 25%) and mobile phones (5/32, 16%). Formal carers in the photo-activity group sometimes used more than one device during the intervention period, which is accounted for in the total devices used (N=32).

**Table 4:** Results regarding implementation factors for formal carers in the photo activity (experimental) and general conversation (control) groups based on comparable questions from the semistructured interviews after the 4-week intervention.

Outcome variable		Experimental group (n=18), n (%)	Control group (n=24), n (%)	Difference tests				*P* value^a^		Effect size			
					*U*		Chi-square (*df*)				*r*		Cramér V
**Medium and format training**					209.5		—^b^		.83		−0.03		—
	Very effective	2 (11)	1 (4)										
	Effective	12 (67)	20 (83)										
	Somewhat effective	3 (17)	2 (8)										
	Not effective	1 (6)	1 (4)										
**Activity duration^c^**					—		0.4 (2)		.84		—		0.09
	Too short	1 (4)	2 (7)										
	Just right	13 (56)	13 (48)										
	Too long	9 (39)	11 (41)										
	Missing	0 (0)	1 (4)										
**4-wk activity period**					—		1.1 (2)		.59		—		0.16
	Too short	2 (11)	1 (4)										
	Just right	9 (50)	15 (62)										
	Too long	7 (39)	8 (33)										
**Activity frequency**					—		0.8 (2)		.67		—		0.14
	Too little	0 (0)	1 (4)										
	Just right	10 (56)	12 (50)										
	Too often	8 (44)	11 (46)										

^a^Significance level set at *P*<.05.

^b^Not applicable.

^c^The n varies for Activity duration, as some formal carers were asked to answer this question multiple times if they did the photo-activity or control activity with more than one resident. Thus formal carers in the photo-activity is n=23, and control activity n=27, as in Figure 4.

#### Mechanism-of-Impact Factors

The majority of residents in the photo activity group (25/29, 86%) reported that they would like to continue participating in the activity with their formal carers. The residents gave the Fotoscope app a score of 8.6 out of 10 in terms of enjoyment. Thematic analysis revealed that the residents in the photo activity group responded more positively when asked whether they wanted to continue performing the activity, especially if new photos were introduced.

A large majority of the formal carers in the photo activity group reported that the reaction of the resident to the photo activity was “very positive” (10/23, 43%) or “positive” (8/23, 35%). The formal carers in the control group predominantly reported that the reaction of the resident to the general conversation activity was “positive” (12/27, 44%), while a minority reported that the reaction was “very positive” (5/27, 19%). Table 5 presents the results from the statistical analysis of differences between residents (in terms of feeling heard, feeling better known by their carers, and their experience of the general conversation), informal carers (whether they heard anything back from the resident about the activity), and formal carers (whether they thought the activity was enjoyable for the resident, was a good way to get to know the resident better, and how much better they got to know the resident from the activity) in the photo activity and control groups.

There was a significant difference between the photo activity and control groups in terms of how the residents felt about having conversations with their formal carers, with a medium effect size (*U=*533.0, *z=*2.865, *r*=0.39; *P=*.004), favoring the photo activity group (median 4.00, “very nice,” n=28) over the control group (median 3.00, “nice,” n=27).

There was also a significant difference in how much better the formal carers in the photo activity group (median 2, “yes, a little better,” n=22) got to know the resident in the past month compared to those in the control group (median 2, “yes, a little better,” n=27; *U*=390.5, *z*=2.114, *r*=0.302 [medium effect]; *P*=.04). The answer options in the semistructured interviews for the photo activity group were as follows: 1=no, not better; 2=yes, a little better; and 3=yes, much better. As the answer options for this question for the control group differed (an additional answer option of “yes, better” was provided after “yes, a little better”), it was decided to combine the answers “yes, better” and “yes, a little better” from the control group interviews in the analysis. Of the formal carers in the experimental group, 48% (11/23) and 39% (9/23) reported getting to know the resident with dementia “a little better” and “much better,” respectively (compared to 17/27, 63% and 4/27, 15%, respectively, of the formal carers in the control group). Of the formal carers in the experimental group, 9% (2/23) reported that they did not get to know the person with dementia “better” compared to 22% (6/27) of the formal carers in the control group. Thematic analysis reflected similar findings: formal carers in both the photo activity and general conversation groups mentioned learning more about the residents:

During care, of course we chat plenty but here I went a bit deeper into the past, so yes I did get to know her much better than I already did.

Photo activity group, formal carer 201

I now know a little more about her past, as far as she could still tell.

Control group, formal carer 204

However, a formal carer in the control group did mention that the general conversation activity was similar to what she already did as part of her work:

Personally it has no added value for me. This is what I do daily in my work—speak to residents every day.

Formal carer 116

Finally, the carers in the photo activity group (n=23) gave a significantly higher rating to the activity (median 9.00) compared to the carers in the general conversation group (n=27; median 8.00) as a way to get to know the resident with dementia better (*U*=419.0, *z*=2.169, *r*=0.307 [medium effect size]; *P*=.03).

There was no statistically significant association between the experimental and control groups on whether residents felt heard during the conversation with their formal carer, whether informal carers received feedback from the residents about the activity, and how much formal carers felt the residents enjoyed the activities (Table 5).

**Table 5:** Results regarding the mechanism-of-impact factors for residents, informal carers, and formal carers in the photo activity (experimental) and general conversation (control) groups based on comparable questions from the semistructured interviews after the 4-week intervention.

Outcome variable				Experimental group	Control group					Difference tests									*P* value^a^			Effect size			
										Fisher exact test			Chi-square (*df*)			*U*						φ			*r*
**Residents, n/N (%)**																									
	Feeling heard: yes			26/29 (90)			24/29 (83)		—^b^			—			—			.49			—			—	
	**Felt better known by their carer**									—			0.3^c^ (1)			—			.60			−0.12			—
		A lot better or better	17/29 (59)			15/29 (52)																			
		A little better or not better	6/29 (21)			9/29 (31)																			
		Missing	6/29 (21)			5/29 (17)																			
	**Experience of general conversation**									—			—			533.0			.004			—			0.39
		Very nice	16/29 (55)			6/29 (21)																			
		Nice	11/29 (38)			15/29 (52)																			
		Somewhat nice	0/29 (0)			5/29 (17)																			
		Not nice	1/29 (3)			1/29 (3)																			
		Missing	1/29 (3)			2/29 (7)																			
**Informal carers, n/N (%)**										—			—			—			—			—			—
	Resident talked to them about the activity: yes			7/28 (25)			5/27 (19)																		
**Formal carers**										—			—			264.0			.20			—			.20
	Enjoyable for people with dementia in a nursing home (0-10), median (IQR)			8.00 (7-9)			8.00 (7-8)																		
	**Knew resident better, n/N (%)**									—			—			390.5			—			—			.30
		Yes, much better	9/23 (39)			4/27 (15)											.04								
		Yes, a little better	11/23 (48)			17/27 (63)																			
		No, not better	2/23 (9)			6/27 (22)																			
		Missing	1/23 (4)			0/27 (0)																			
		Way of getting to know resident with dementia better (0-10), median (IQR)	9.00 (7-9)			8.00 (6-8)		—			—			419.0			.03			—			0.31		

^a^Significance level set at *P*<.05.

^b^Not applicable.

^c^Chi-square test with Yates continuity correction.

#### Experienced Usability

In the thematic analysis, the formal carers in the photo activity group described the Fotoscope app as easy to use, simple, clear, and visually appealing. Technical issues were experienced (eg, difficulties logging in or the app not working on the tablet). However, carers mentioned that the Fotoscope app enhanced social interaction, helping them to get to know the resident better:

It triggers more conversation, especially for the quieter ones, this can work up to a conversation.

Formal carer 201

#### Experienced Usefulness

The majority of the formal carers in the photo activity group found the preparation for the photo activity useful (14/18, 78%) or very useful (2/18, 11%), while a small minority found it not useful (2/18, 11%). Whereas the majority of the formal carers in the photo activity group found the black-and-white photos “pleasant” (4/18, 22%) or “neutral” (7/18, 39%), some found them “unpleasant” (7/18, 39%). A number of formal carers in the photo activity group noted that some photos would really have been better with color:

I was sorry we didn’t have color photos. Some things really needed color. Even my client asked me why I didn’t have color photos.

Formal carer 105 with resident 109

However, formal carers also mentioned that while they preferred color photos, residents did not mind viewing black-and-white photos:

I would personally like it better if the pictures were in color, but for the resident this did not matter.

Formal carer 105 with resident 121

[T]hey [residents] mostly liked black-and-white pictures too.

Formal carer 101

The number of photos in the database was identified as a subtheme in the thematic analysis. While the formal carers described the selection as varied, some noted a lack when residents had specific interests; for example, a resident wanted to see more ballet pictures.

Google Analytics showed that while all users viewed the Profile page, 19% (4/21) did not add photos to the Photo Selection page, and only 48% (10/21) used the photo selection tool on the Profile page to select photos. In addition, while the majority of users (17/21, 81%) opened photos from the Photo Selection page as instructed, 19% (4/21) apparently accessed photos through other means. Furthermore, only 33% (7/21) of the users used the Favorites page.

#### Learnability

The majority of formal carers in the photo activity group (12/18, 67%) said that it was easy to learn how to use the Fotoscope web application. Google Analytics showed that the most viewed page of the main pages was the Profile page (257/594, 43.3% total unique page views), followed by the general Themes page (104/594, 17.5%), Favorites page (85/594, 14.3%), “joker” page (57/594, 9.6%), and Search (43/594, 7.2%). The User Guide (29/594, 4.9%) and Information page (19/594, 3.2%) were the least viewed pages. The most frequently selected main photo category (based on Google Analytics’ unique events count) was people (71/404, 17.6%), followed by activities (64/404, 15.8%), and places and animals (both 56/404, 13.9%). The least selected main photo categories were experiences (53/404, 13.1%), nature (53/404, 13.1%), and things (51/404, 12.6%).

#### Adoption

The majority of formal carers in the photo activity group (14/18, 78%) agreed that the Fotoscope app can be used as part of daily care activities in the nursing home.

## Discussion

### Principal Findings and Comparisons With Prior Work

#### Overview

The goal of this study was to determine contextual, implementation, and mechanism-of-impact factors [17] that may have facilitated or hindered the implementation of the photo activity intervention for residents with dementia and potentially influenced the intervention outcomes.

To answer the RQs, we conducted a mixed methods process evaluation alongside a pilot RCT investigating the feasibility and effectiveness of the photo activity for residents and their informal and formal carers. The main findings of this process evaluation were as follows. For contextual factors affecting the implementation of the intervention (RQ1), formal carers struggled to find the time to conduct the activity during their work hours. For implementation factors, which looked at how users assessed the preparation, introduction, and delivery of the interventions (RQ2), it was found that viewing digital photos on a tablet was feasible for residents with dementia; formal carers from both groups were mostly positive and satisfied regarding the online training sessions but suggested that, in the future, they would like shorter sessions as well as in-person training and support; and informal carers would like more updates from formal carers. For the mechanisms of impact (RQ3), looking into how users experienced and valued the digital photo activity versus the control activity, it was found that residents who performed the digital photo activity with their carers had a more positive experience compared to residents in the control activity. Formal carers who conducted the digital photo activity reported getting to know the residents “much better” compared to formal carers in the control activity. The Fotoscope app as a tool to deliver the digital photo activity was also rated as a better way to get to know the residents as opposed to having a general conversation. Informal carers of residents in the photo activity group also reported more contact with formal carers compared to informal carers in the control activity.

In the following subsections, we provide a more detailed discussion of the findings in relation to each RQ.

#### How Did Factors External to the Intervention Affect the Implementation of the Intervention (Context)?

While minimal contextual factors impeded the implementation of the photo activity or the control activity, a salient theme that emerged was the difficulty that formal carers experienced in scheduling time to conduct the activities with the resident alongside their daily care tasks. In some cases, formal carers mentioned having to conduct the activity outside of their work shift. Problems with workload and staffing are common barriers to implementing psychosocial interventions successfully in nursing homes [14]. Staff are pressured to prioritize tasks that are related to physical and safety needs [35]. Due to the restrictions on visiting nursing homes during the COVID-19 pandemic [36], during the initial phase of the study, no informal carers were involved in delivering either activity to residents. Later, no informal carers were willing to invest the time needed to provide the intervention or control activity.

It is not clear which device carers used to prepare for the photo activity and which device they used to conduct the activity; therefore, it is possible that carers used a desktop computer to prepare and select initial photos for the activity and then used the tablet as instructed during the activity with the resident.

#### How Did Residents and Their Informal and Formal Carers Assess the Preparation, Introduction, and Delivery of the Interventions (Implementation)?

Most residents responded positively to viewing photos on a tablet, consistent with literature suggesting that using tablets in art-based psychosocial interventions could have positive effects for individuals with dementia [37,38].

Informal carers from both groups mentioned wanting more updates from formal carers on how the residents were responding to the activities. A previous study found that greater involvement of informal carers in the daily care of residents was an important factor in the successful implementation of psychosocial interventions because this also helps formal carers to become more familiar with the residents and their identity within a family context [39]. The communication between informal and formal carers fosters more trust, which benefits overall care [14].

Regarding the online training sessions, while the majority of the formal carers from both groups were positive and satisfied, formal carers from both groups who were dissatisfied with the training sessions suggested shortening the duration and expressed a preference for live training and in-person support or demonstration. The setting of the nursing home itself may also have influenced how staff received the training. Kuske et al [40] found that when staff feel overburdened, they see training as an additional task rather than beneficial continuing education, which could explain why shortening the duration of the training sessions was a salient theme in this process analysis.

A minority of formal carers who conducted the photo activity with residents with a more severe level of dementia (ie, a GDS score of 5 or 6) [23] described the digital photo activity as not suitable and the 30-minute activity duration as too long for these residents due to their “short attention spans” or because “they get tired easily.” This is similar to previous work suggesting that residents’ deteriorating health could be a barrier for formal carers implementing psychosocial interventions [40].

However, it is worth noting that the majority of the residents had a GDS score of 5 or 6 [23], and the experience of most formal carers was that the photo activity intervention was easy to implement and that the activity duration was just right. This suggests that the photo activity can be a suitable addition to tools that nursing homes could use, including for residents with more severe dementia, because most interventions for people with more severe dementia are focused on reducing problem behaviors rather than improving social interactions or quality of life [41,42].

Some residents experienced difficulty in responding to the SFAS [30], which presented 5 emotions or faces. Future trials could explore using a simpler scale similar to the SFAS but with only 3 faces (happy, neutral, and sad) because a study found that older adults sometimes have difficulty differentiating between “very happy” and “happy” and between “very sad” and “sad” [43].

Google Analytics may not have accurately recorded actual use of the app by formal carers for the process evaluation, as shown by the lack of correlation between self-reported use and observed use via Google Analytics. Currently, photos can be viewed in the Fotoscope web application via the Themes page without logging in; therefore, it is possible that formal carers may not have logged in during some of the sessions and manually searched for photos instead of accessing the Profile page or Photo Selection page. For future trials, it could be useful to consult with software and app developers and explore integrating an in-app analytics function within the Fotoscope app rather than relying on an external analytics provider.

#### How Did the Residents With Dementia and Their Informal and Formal Carers Experience and Value the Digital Photo Activity Versus the General Conversation Activity (Mechanisms of Impact)?

Residents in the photo activity group reported a more positive experience engaging in the activity with their formal carers. The positive findings regarding using generic photos in the photo activity are in line with a similar study that used generic photos as conversation prompts for people with dementia, where it was found that compared to using family photos, people with dementia told more detailed and personally relevant stories when shown generic photos [42].

More residents in the photo activity group were eager to continue the activity with their formal carers. This highlights the importance of investing in interventions that foster social health for residents with dementia in the nursing home, rather than solely focusing on physical needs or problematic behaviors [41,44].

Informal carers in the photo activity group had more contact with the formal carers, and more residents in the photo activity group talked to their informal carers about the activity. This finding shows that the photo activity may be able to enhance the involvement of informal carers, a factor that is crucial to consider in the successful implementation of psychosocial interventions [14,39].

The formal carers in the photo activity group perceived the residents’ reaction to the conversation about the photos as more positive. These carers also reported getting to know the residents much better and rated the Fotoscope app higher (compared to a general conversation) in terms of using it as a tool to get to know the resident better. This is in line with the findings of Lawrence et al [14] indicating that when formal carers feel that an intervention helps them develop a more personal relationship with a resident and that the intervention is meaningful for the resident, they are more likely to continue using the intervention because they see that it has value. This is further supported by the findings from the process evaluation: whereas a formal carer mentioned that the control activity had no added value for her, the majority of formal carers in the photo activity group felt that the intervention could be adopted into daily care because of its value.

The Fotoscope web application was generally well received and was described as having a simple and pleasing design and user interface, which is crucial for engaging users of technological interventions [6,45]. More formal carers were vocal about the black-and-white photos compared to the residents, but only a minority of the formal carers found the black-and-white photos unpleasant. Previous studies have shown that both color and black-and-white photos elicit reactions from residents with dementia equally [42,46]. Residents who participated in the photo activity were also observed to react to the black-and-white photos in the Fotoscope app and mostly found the photos pleasant or neutral.

### Strengths and Limitations

This study has a number of strengths. First, data were collected from nursing home residents, informal carers, and formal carers, providing a wider understanding of the process of implementing the intervention from all viewpoints as well as the contextual and mechanism-of-impact factors that potentially influenced the outcomes of the RCT. Mixed methods were used; quantitative and qualitative data were collected from semistructured interviews with participants, and objective data were obtained from Google Analytics. The semistructured interviews were structured according to the MRC guidance for process evaluations of complex interventions [17]. The digital protocol enabled research assistants to conduct interviews with the residents via video calls, ensuring data collection despite the lockdowns and visiting limitations during the COVID-19 pandemic.

Some methodological limitations included changing the frequency of the interventions after the trial had already been running for 1 month after receiving feedback from the first round of formal carers delivering the interventions. Google Analytics data were available for only 21 (51%) of the 41 users (representing carer-resident pairs) because the researchers were unaware and not informed of the data reaching the storage time limit and being deleted from the account before data collection concluded and data analysis started. The manner of collecting responses for the semistructured interviews with carers varied: some carers answered the interview questions on their own, while others preferred to be interviewed over the phone, which may have influenced their answers differently. In relation to the manner of collecting responses for the interviews, not all closed-ended questions invited an elaborate response. This was done keeping in mind the time constraints and effort required from participants. However, the trained research assistants were instructed to write down any answer that may have been given in place of or in addition to the answers to the closed-ended questions, which enabled us to learn, for example, that residents in the photo activity group were eager to continue performing the activity with their carers with new photos.

Finally, we were unable to request feedback from the participants regarding the results of the thematic analysis. Unfortunately, no informal carers were involved in delivering the control or experimental interventions, despite recruitment efforts aimed at future implementation. As a result, no data could be collected on implementation or mechanism-of-impact factors from informal carers providing the photo activity intervention.

### Scientific Relevance of the Study and Recommendations for Future Studies

The mixed methods process evaluation showed that despite the complexity of the intervention, the digital photo activity was feasible to implement via online training sessions and off-site monitoring, providing a good basis for future larger-scale research on the intervention. The MRC guidance was useful in identifying contextual, implementation, and mechanism-of-impact factors that positively or negatively affected implementation and potentially influenced the intervention outcomes. It is recommended that a definitive trial investigating the effectiveness of the intervention should implement feedback from this process evaluation, such as in-person staff training and demonstration of the Fotoscope app. Informal carers could also be recruited to carry out the photo activity, now that COVID-19 restrictions are no longer in place, to explore what implementation issues would be in play for them and how they could benefit from offering the intervention. Incorporating an automatic record of activity appointments in the Fotoscope app instead of asking users to record their time and date of use manually might also provide a more accurate account of their use of the web application.

### Clinical Relevance of the Study and Implications for Practice

The mixed methods process evaluation found that residents participating in the photo activity enjoyed it more than those participating in the general conversation activity. This confirms a previous finding that touchscreen art interventions could be beneficial for residents’ psychological well-being [6]. Ensuring that tablets are easily accessible in the wards is recommended to encourage staff to conduct the photo activity. While it is noted that the control condition did not include any art-related components, part of the aims of this process evaluation was investigating how the photo activity intervention compared to an active control activity that was most likely to happen naturally within the context of the nursing home (in this case, sitting down with a resident for a chat over a warm drink compared to setting up a different art activity).

Formal carers in the photo activity group got to know the resident with dementia substantially better and rated the photo activity as a better way of getting to know the resident. This finding is relevant because previous literature has found that a more person-oriented care for people living with dementia leads to better outcomes associated with social health and quality of life [29,47], and the photo activity could be an easy and meaningful activity to help formal carers to get to know the residents better. In addition, the importance of formal carers communicating with the informal carers can be encouraged as an essential part of the photo activity.

A majority of the photo activity carers agreed that the intervention could be part of daily care activities, but important contextual factors such as heavy workload and a lack of staff need to be taken into account [14]. Involving the wider organization is important to allow formal carers to view psychosocial interventions as part of daily care tasks, rather than an added workload [48]. For the photo activity, supporting staff to conduct the intervention during their shifts, not outside of paid hours, is recommended, as well as being flexible with the activity duration.

### Conclusions

This study showed that the initial training and preparation for formal carers in both the photo activity and control groups, as well as their perception of effort and time investment, did not seem to differ much. The findings suggest that implementing the photo activity as an intervention does not require a significant amount of additional work, yet it offers notable benefits, including residents enjoying the photo activity more and formal carers getting to know the residents better. Therefore, it is suggested that the digital photo activity, as an enjoyable intervention with its easy to use web application, could be widely implemented and disseminated in nursing homes. As a person-centered intervention designed to enhance relationships among people with dementia in nursing homes and their informal and formal carers, it may have the potential to positively impact outcomes relating to social health and quality of life of the person with dementia, but this needs to be explored further.

## Appendix

Multimedia Appendix 1 Topics for each Medical Research Council guidance for process evaluation component and the measurements used.

Multimedia Appendix 2 Responses to closed-ended questions from the semistructured interviews with residents and carers.

Multimedia Appendix 3 Thematic analysis themes, subthemes, and sample data extracts.

## Abbreviations

COREQ: Consolidated Criteria for Reporting Qualitative Research
GDS: Global Deterioration Scale
GRAMMS: Good Reporting of a Mixed Methods Study
MRC: Medical Research Council
RCT: randomized controlled trial
RQ: research question
SFAS: Smiley Face Assessment Scale
