# Supplementary material for: A Digital Photo Activity Intervention for Nursing Home Residents With Dementia and Their Carers: Mixed Methods Process Evaluation
Source: JMIR Form Res. 2025 Apr 16;9:e56586. doi: 10.2196/56586 (PMC12044310; doi:10.2196/56586)
Supplement: Multimedia Appendix 1 [file formative_v9i1e56586_app1.docx]

Supplementary File 1. Topics for each MRC component and measurements used

| **MRC Component** | | **Topics** | | | **Measurement Used** | | | **Participant** | | | | | |  |  |  |  |  |  |  |
| --- | --- | --- | --- | --- | --- | --- | --- | --- | --- | --- | --- | --- | --- | --- | --- | --- | --- | --- | --- | --- |
|  | Participant demographics | | | | | Demographic questionnaire (based on TOPICS-MDS) | | | | | | All | | | | | | |  |  |
| Contextual | Prior tablet use | | | | | Semi-structured interview form | | | | | | (In)Formal carer | | | | | | |  |  |
| Factors | Frequency of using tablet vs other devices | | | | | Google Analytics | | | | | | (In)Formal carer | | | | | | |  |  |
|  | Material, social, and/or financial circumstances influencing being able to do the activities with the residents | | | | | Semi-structured interview form | | | | | | (In)Formal carer | | | | | | |  |  |
|  | Feedback on space where resident did activity with carer | | | | | Semi-structured interview form | | | | | | Resident | | | | | | |  |  |
| Implementation Factors | Amount and clarity of information, resources and support | | | Semi-structured interview form | | | | | | | | | (In)formal carer | | | | |  |  |  |
|  | Experience of online training and introductory sessions | | | | | Semi-structured interview form | | | | | (In)Formal carer | | | | | |  |  |  |  |
|  | Viewing black and white digital photos on tablet | | | | | Semi-structured interview form | | | | | Resident, (In)Formal carer | | | | | |  |  |  |  |
|  | Feasibility of activity duration | | | | | Semi-structured interview form, Google Analytics, Carer Self-report Registration | | | | | (In)Formal carer | | | | | |  |  |  |  |
|  | Doing the activity in the nursing home | | | | | Semi-structured interview form | | | | | (In)Formal carer | | | | | |  |  |  |  |
|  | Participating in video-call observations and receiving feedback from research assistant | | | | | Semi-structured interview form | | | | | (In)Formal carer | | | | | |  |  |  |  |
|  | Feasibility of using SFAS for resident with dementia | | | | | Semi-structured interview form | | | | | (In)Formal carer | | | | | |  |  |  |  |
|  | Technical problems (with Fotoscope) | | | | | Semi-structured interview form | | | | | (In)Formal carer | | | | | |  |  |  |  |
| Mechanisms of  Impact | Experience of the conversation during the activities | | | | | Semi-structured interview form | | | | | Resident, (In)Formal carer | | | | | |  |  |  |  |
|  | Perception on how well photos matched interest | | | | | Semi-structured interview form | | | | | Resident, (In)Formal carer | | | | | |  |  |  |  |
|  | Experience of using the Fotoscope app | | | | | Semi-structured interview form | | | | | Resident, (In)Formal carer | | | | | |  |  |  |  |
|  | Feasibility of activity period and frequency | | | | | Semi-structured interview form | | | | | (In)Formal carer | | | | | |  |  |  |  |
|  | Perceived reaction of person with dementia to the activity | | | | | Semi-structured interview form | | | | | (In)Formal carer | | | | | |  |  |  |  |
|  | How much better did carers get to know the resident | | | | | Semi-structured interview form | | | | | Formal carer | | | | | |  |  |  |  |
|  | Resident’s feeling of being known and heard by their carer | | | | | Semi-structured interview form | | | | | Resident | | | | | |  |  |  |  |
|  | Perceived usefulness of the activity for the resident | | | | | Semi-structured interview form | | | | | Informal carer | | | | | |  |  |  |  |
|  | Frequency of hearing about the activity from the resident | | | | | Semi-structured interview form | | | | | Informal carer | | | | | |  |  |  |  |
| *Experienced usability* | Fotoscope's ease of use·  Impression of Fotoscope app; likes and dislikes | | | | | Semi-structured interview form  Semi-structured interview form | | | | | (In)Formal carer  (In)Formal carer | | | | | |  |  |  |  |
| *Experienced usefulness* | Experience of practical preparation for the Fotoscope | | | | | Semi-structured interview form,  Google Analytics | | | | (In)Formal carer | | | | |  |  |  |  |  |  |
|  | Use of Fotoscope pages/functions | | Semi-structured interview form  , Google Analytics | | | | | | | (In)Formal carer | | | | | | | | | | |
| *Experienced*  *learnability* | Experience of learning to use the Fotoscope | | | Semi-structured interview form, Google Analytics | | | | | | (In)Formal carer | | | | | | |  |  |  |  |
|  | Use of Information and User Guide pages in Fotoscope | | | Semi-structured interview form, Google Analytics | | | | | (In)Formal carer | | | | | | | | | | |  |
| *Adoption* | Perception of using Fotoscope app in daily care in nursing home | | | | | | Semi-structured interview form | | Formal carer | | | | | | |  |  |  |  |  |
