# Supplementary material for: A Digital Photo Activity Intervention for Nursing Home Residents With Dementia and Their Carers: Mixed Methods Process Evaluation
Source: JMIR Form Res. 2025 Apr 16;9:e56586. doi: 10.2196/56586 (PMC12044310; doi:10.2196/56586)
Supplement: Multimedia Appendix 2 [file formative_v9i1e56586_app2.docx]

Supplementary File 2. Semi-structured interview questions and answer summaries for closed questions

| **Residents** | Experimental % (n=29) | Control % (n=29) |
| --- | --- | --- |
| **Contextual Factors** |  |  |
| *How do you feel about the place or space to view the photos together and have a conversation (Photo-activity)/ to have a conversation (control)* |  |  |
| Comfortable, quiet enough, peaceful | 79.3% (23) | 93.1% (27) |
| Not comfortable, noisy, restless | 3.4% (1) | 0 |
| Missing | 17.2% (5) | 6.9% (2) |
| **Implementation Factors** |  |  |
| *You have now done the Photo-Activity together several times. Do you find it going well with the tablet/screen?* |  |  |
| Yes | 86.2% (25) | n/a |
| Sometimes | 3.4% (1) |  |
| No | 0 (0) |  |
| Missing | 10.3% (3) |  |
| *Could you see the photos clearly? (Do you find them sufficiently large and sharp?)* |  |  |
| Yes | 72.4% (21) | n/a |
| Sometimes | 17.2% (5) |  |
| No | 6.9% (2) |  |
| Missing | 3.4% (1) |  |
| *How do you feel about the number of photos you and your carer viewed?* |  |  |
| Too little | 10.3% (3) | n/a |
| Good | 79.3% (23) |  |
| Too much | 3.4% (1) |  |
| Missing | 6.9% (2) |  |
| *How did you feel about the pictures being in black and white?* |  |  |
| Pleasant | 27.6% (8) | n/a |
| Neutral | 48.2% (14) |  |
| Unpleasant | 13.8% (4) |  |
| Missing | 10.3% (3) |  |
|  |  |  |
| **Impact Mechanisms**  *How did you feel about having conversations with your carer about the photos? (Photo-activity)/ How did you find having conversations with your carer (control)* |  |  |
| Not nice | 3.4% (1) | 3.4% (1) |
| A little nice | 0 (0) | 17.2% (5) |
| Nice | 37.9% (11) | 51.7% (15) |
| Very nice | 55.2% (16) | 20.7% (6) |
| Missing | 3.4% (1) | 6.9% (2) |
|  |  |  |
| *Follow-up question: Would you like to continue having these kinds of conversations?* |  |  |
| Yes | 86.2% (25) | 65.5% (19) |
| No | 6.9% (2) | 20.7% (6) |
| Missing | 6.9% (2) | 13.8% (4) |
| *Did the photos match your interest?* |  |  |
| Always | 27.8% (8) | n/a |
| Often | 48.3% (14) |  |
| Sometimes | 10.3% (3) |  |
| Never | 13.8% (4) |  |
| *Did you feel there was enough opportunity to talk about the pictures, and tell what the pictures evoked in you? (Photo-activity)/ Did you feel there was enough opportunity to talk about your thoughts and feelings during the conversation? (control)* |  |  |
| Always | 48.3% (14) | 34.5% (10) |
| Often | 27.6% (8) | 31.0% (9) |
| Sometimes | 0 (0) | 13.8% (4) |
| Never | 0 (0) | 6.9% (2) |
| Missing | 24.14% (7) | 13.8% (4) |
|  |  |  |
| *Follow-up question: Did you feel heard (seen and taken seriously) during the conversation?* |  |  |
| Yes | 89.7% (26) | 82.8% (24) |
| No | 0 (0) | 3.4% (1) |
| Missing | 10.3% (3) | 13.8% (4) |
| *What grade would you give the Fotoscope app if you think about how much fun you had with it ? Enjoyed it? (from 0 = no pleasure at all to 10 very much pleasure)* |  |  |
| M (SD) | 8.6 (1.08) | n/a |
|  | Missing: 14% (4) |  |
| *Do you think your healthcare provider got to know you better by having regular conversations with you about the photos/general topics?* |  |  |
| Much Better | 24% (7) | 3.4% (1) |
| Better | 34.5% (10) | 48.2% (14) |
| A little better | 13.5% (4) | 20.7% (6) |
| No, not better | 6.9% (2) | 10.3% (3) |
| Missing | 20.7% (6) | 17.2% (5) |

| **Informal Carers** | Experimental % (n=28) | Control % (n=27) |
| --- | --- | --- |
| **Contextual Factors** |  |  |
| *Were there any obstacles that prevented you from talking to your loved one's caregiver about your loved one's interests or experiences with the photo activity/ conversational activity (control)?* |  |  |
| Lack of time | 25.0% (7) | 3.7%(1) |
| Language Barrier | 0 | 0 |
| No Communication Device Available | 0 | 0 |
| Vacation | 0 | 3.7%(1) |
| Other | 14.3%(4) | 37.0%(10) |
| None | 46.4%(13) | 55.6%(15) |
| Missing | 14.3% (4) | 0 |
| **Implementation Factors** |  |  |
| *Do you feel you were well informed about the Photo Activity/ conversation (control) activity with your loved one?* |  |  |
| Yes | 89.2% (25) | 74.1%(20) |
| No | 10.7% (3) | 22.2% (6) |
| Missing | 0 | 3.7% (1) |
| *Follow-up questions:* |  |  |
| *Were the information letter and brochures clear?* |  |  |
| Not clear | 0 | 0 |
| Somewhat clear | 17.9% (5) | 11.1%(3) |
| Clear | 60.7% (17) | 62.3% (17) |
| Very clear | 7.1% (2) | 18.5%(5) |
| Missing | 0 | 7.4%(2) |
| *Were purpose and frequency of the activity clear (2x/week 30 min)?* |  |  |
| Not clear | 3.6% (1) |  |
| Somewhat clear | 17.86% (5) | 18.5%(5) |
| Clear | 67.86% (19) | 59.3%(16) |
| Very clear | 7.14% (2) | 11.1%(3) |
| Missing | 3.6% (1) | 11.1%(3) |
| *Did you miss any information?* |  |  |
| No | 67.9% (19) |  |
| Yes | 21.4% (6) | 22.2%(6) |
| Missing | 10.7% (3) | 74.1%(20) |
| **Impact Mechanisms** |  |  |
| *How did you feel about the conversation with the caregiver about your loved one's preferences and interests?* |  |  |
| Not useful | 0 | n/a |
| A little useful | 10.7% (3) |  |
| Useful | 57.1% (16) |  |
| Very useful | 10.7% (3) |  |
| Missing | 21.4% (6) |  |
| *Were the right questions asked?* |  |  |
| Yes | 71.4% (20) | n/a |
| No | 7.1% (2) |  |
| Missing | 21.4% (6) |  |
| *Did you feel heard?* |  |  |
| Yes | 75.0% (21) | n/a |
| No | 7.1% (2) |  |
| Missing | 17.9% (5) |  |
| *How did you feel about your loved one doing the conversation activity (control) with a caregiver from the nursing home?* |  |  |
| Not nice | n/a | 0 |
| Somewhat nice |  | 3.7%(1) |
| Nice |  | 44.4%(12) |
| Very nice |  | 29.6%(8) |
| Missing |  | 22.2%(6) |
| *In the past month, has your loved one mentioned anything to you about the Photo Activity/ conversation activity (control) with the caregiver during your visits/phone calls?* |  |  |
| No, nothing | 71.4% (20) | 81.5%(22) |
| Yes | 28.6% (8) | 18.5%(5) |
| Missing | 0 |  |
| *Has your loved one's caregiver been in contact with you in the past month about how the Photo Activity/ Conversation activity (control) was experienced by your loved one (e.g., by phone, email, or while visiting your loved one)?* |  |  |
| No, never | 32.1% (9) | 51.9%(14) |
| One time | 32.1% (9) | 22.2%(6) |
| Multiple times | 21.4% (6) | 22.2%(6) |
| Every week | 7.1% (2) | 0 |
| Missing | 7.1% (2) | 3.7%(1) |

| **Formal Carers** | Experimental (n=18) | Control (n=24) |
| --- | --- | --- |
| **Contextual Factors** |  |  |
| *Did you have experience using a tablet before participating in this study?* | n=18 |  |
| Yes, a lot | 55.6% (10) | n/a |
| Yes, a little | 27.8% (5) |  |
| Hardly | 5.6% (1) |  |
| No, not at all | 11.1% (2) |  |
| Missing |  |  |
| *Did material, social and/or financial (work) circumstances influence your use, or frequency of use, of the Fotoscope app in any way?* |  |  |
| *- For material circumstances, think e.g.: was a tablet available (in time), was there a quiet place where you could do the Photo activity with the person with dementia, etc.?* |  |  |
| *- For social circumstances, consider e.g.: did your manager allocate you time to participate in the project, etc.?* |  |  |
| *- For financial circumstances, consider e.g. budget cuts, resulting in less manpower available in department* | n=18 |  |
| No | 77.8% (14) | n/a |
| Yes | 22.2% (4) |  |
| Missing | 0 |  |
| *Did material, social and/or financial (work) circumstances influence you being able to do the general Conversation activity with the resident in any way?* |  |  |
| *- For material circumstances, think e.g.: was there a quiet place where you could do the activity with the person with dementia, etc.?* |  |  |
| *- For social circumstances, think e.g.: did your manager allocate you time to participate in the project, etc.?* |  |  |
| *- For financial circumstances e.g. think about budget cuts, which meant less human resources were available in department* |  | n=24 |
| No | n/a | 62.5% (15) |
| Yes |  | 37.5% (9) |
| *Missing* |  | 0 |
| **Implementation Factors** |  |  |
| *What did you think of the online Introduction and Training session for the Photo Activity and the Fotoscope app? (1 hour)* | n=18 |  |
| Unsatisfactory | 11.1% (2) | n/a |
| Moderate | 16.7% (3) |  |
| Satisfactory | 44.4% (8) |  |
| Good | 27.8% (5) |  |
| Missing | 0 |  |
| *What did you think of the online Introduction and Training session for the general conversation activity (30-45 minutes)* |  | n= 24 |
| Unsatisfactory | n/a | 0 |
| Moderate |  | 8.3% (2) |
| Satisfactory |  | 58.3% (14) |
| Good |  | 33.3% (8) |
| Missing |  | 0 |
| *Follow-up questions:* |  |  |
| *How did you find the length of the online training?* | n=18 | n=24 |
| Too long | 33.3% (6) | 12.5% (3) |
| Just right | 66.7% (12) | 83.3% (20) |
| Too short | 0 | 4.2% (1) |
| Missing | 0 | 0 |
| *Was there sufficient opportunity to ask questions?* | n=18 | n=24 |
| Yes | 94.4% (17) | 91.7% (22) |
| No | 5.6% (1) | 8.3% (2) |
| Missing | 0 | 0 |
| *What did you think of the medium and format (via Microsoft Teams, a mix of live and recorded speakers)?* | n=18 | n=24 |
| Not effective | 5.6% (1) | 4.2% (1) |
| Little effective | 16.7% (3) | 8.3% (2) |
| Effective | 66.7% (12) | 83.3% (20) |
| Very effective | 11.1% (2) | 4.2% (1) |
| Missing | 0 | 0 |
| *How did you feel about the researchers' support and the materials they provided you with in implementing the intervention?* | n=18 | n=24 |
| Unsatisfactory | 0 | 0 |
| Moderate | 0 | 4.2% (1) |
| Satisfactory | 77.8% (14) | 66.7% (16) |
| Good | 22.2% (4) | 29.2% (7) |
| Missing | 0 | 0 |
| *Follow-up questions:* |  |  |
| *Did you find the resources useful (Training manual, presentations, etc.)?* | n=18 | n=24 |
| Yes | 83.3% (15) | 79.2% (19) |
| No | 11.1% (2) | 8.3% (2) |
| Missing | 5.6% (1) | 12.5% (3) |
| *Was the 30-minute Photo Activity/ conversation activity feasible for the person with dementia?* | n=23 | n=27 |
| Too long | 39.1% (9) | 40.7% (11) |
| Just right | 56.5% (13) | 48.1% (13) |
| Too short | 4.3% (1) | 7.4% (2) |
| Missing | 0 | 3.7% (1) |
| *Could the person with dementia see the pictures on the tablet clearly?* | n=23 |  |
| Yes | 78.2% (18) | n/a |
| No | 21.7% (5) |  |
| Missing | 0 |  |
| *Were you distracted by students attending and observing via video call?* | n=18 | n=24 |
| Yes, I was distracted by it | 0 | 0 |
| No | 100% (18) | 91.7% (22) |
| Missing | 0 | 8.3% (2) |
| *Was the person with dementia distracted by students attending and observing via video call?* | n=23 | n=27 |
| Yes, the person with dementia was distracted by it | 13.0% (3) | 18.5% (5) |
| No | 87.0% (20) | 77.8% (21) |
| Missing | 0 | 3.7% (1) |
| *How did you experience explaining the Photo Activity and the Fotoscope app to the resident/person with dementia?* | n=23 |  |
| Very difficult | 4.3% (1) | n/a |
| Difficult | 17.4% (4) |  |
| Easy | 60.9% (14) |  |
| Very easy | 17.4% (4) |  |
| *How did you experience explaining the Photo Activity and the Fotoscope app to their loved one* | n=23 |  |
| Very difficult | 0 | n/a |
| Difficult | 0 |  |
| Easy | 78.2% (18) |  |
| Very easy | 13.0% (3) |  |
| Missing | 8.7% (2) |  |
| *Could the person with dementia give you an answer on the SMILEY Face Assessment Scale (SFAS) ?* | n=23 | n=27 |
| Yes | 82.6% (19) | 70.4% (19) |
| No | 13.0% (3) | 14.8% (4) |
| Missing | 4.3% (1) | 14.8% (4) |
| *How did you experience the feedback session with the students?* | n=23 |  |
| Not instructive | 8.7% (2) | n/a |
| Somewhat instructive | 13.0% (3) |  |
| Instructive | 65.2% (15) |  |
| Very instructive | 4.3% (1) |  |
| Missing | 8.7% (2) |  |
| *What was your experience of doing the Photo Activity using the Fotoscope, with the person with dementia?* | n=23 |  |
| Difficult to implement | 13.0% (3) | n/a |
| Easy to implement | 69.6% (16) |  |
| Very easy to implement | 13.0% (3) |  |
| Missing | 4.3% (1) |  |
| *Have you encountered any technical problems when using the Fotoscope?* | n=18 |  |
| No | 72.2% (13) | n/a |
| Yes, with the Themes page | 5.6% (1) |  |
| Yes, with the Profile page | 0 |  |
| Yes, with the Favourites page | 0 |  |
| Yes, with the Joker page | 0 |  |
| Yes, with the User Guide page | 0 |  |
| Yes, with the Information page | 0 |  |
| Other | 16.7% (3) |  |
| Missing | 5.6% (1) |  |
| **Impact Mechanisms** |  |  |
| *How do you feel about the photos in Fotoscope being shown in black and white?* | n=18 |  |
| Pleasant | 22.2% (4) | n/a |
| Neutral | 38.9% (7) |  |
| Unpleasant | 38.9% (7) |  |
| Missing | 0 |  |
| *Have you found pictures that match the interests of the person with dementia?* | n=23 |  |
| Yes | 95.7% (22) | n/a |
| No | 4.3% (1) |  |
| Missing | 0 |  |
| *Did you search for specific photos/themes' in Fotoscope that you could not find? In other words, did you miss any photos/themes?* | n=23 |  |
| Yes | 60.9% (14) | n/a |
| No | 39.13% (9) |  |
| Missing | 0 |  |
| *How did you feel about having conversations with the person with dementia about the photos on the tablet (experimental) /general topics (control)?* | n=23 | n=27 |
| Not so nice | 4.3% (1) | 0 |
| A bit nice | 4.3% (1) | 14.8% (4) |
| Nice | 39.1% (9) | 48.1% (13) |
| Very Nice | 52.2% (12) | 37.0% (10) |
| Missing | 0 | 0 |
| *What did you think of the four-week period?* | n=18 | n=24 |
| Too long | 38.9% (7) | 33.3% (8) |
| Just right | 50.0% (9) | 62.5% (15) |
| Too short | 11.1% (2) | 4.2% (1) |
| Missing | 0 | 0 |
| *How did you feel about the frequency: twice a week for half an hour?* | n=18 | n=24 |
| Too often | 44.4% (8) | 41.7% (10) |
| Just right | 55.6% (10) | 54.2% (13) |
| Too little | 0 | 4.2% (1) |
| Missing | 0 | 0 |
| *What was the reaction of the person with dementia about the photos (experimental)/ general conversation (control)?* | n=23 | n=27 |
| Negative | 0 | 0 |
| Neutral | 17.4% (4) | 37.0% (10) |
| Positive | 34.8% (8) | 44.4% (12) |
| Very Positive | 43.5% (10) | 18.5% (5) |
| Missing | 4.3% (1) | 0 |
| *Did the response of the person with dementia match your expectation?* | n=23 | n=27 |
| More negative than expected | 4.3% (1) | 3.7% (1) |
| In line with expectation | 69.6% (16) | 63.0% (17) |
| More positive than expected | 26.1% (6) | 29.6% (8) |
| Missing | 0 | 3.7% (1) |
| *What rating would you give the Fotoscope app in terms of how much you personally enjoyed using it in a conversation with the person with dementia/resident? (from 0 = no enjoyment at all to 10 - very much enjoyment you experienced from it)* | n=18 |  |
| M (SD) | 7.8 (1.26) | n/a |
| *Did you get to know the person with dementia better through the Photo-Activity in the past month?* | n=23 | n=27 |
| No | 8.7% (2) | 22.2% (6) |
| Yes, a little better | 47.8% (11) | 63.0% (17) |
| Yes, much better | 39.1% (9) | 14.8% (4) |
| Missing | 4.3% (1) | 0 |
| **Usefulness** |  |  |
| *How did you experience the practical preparation of the Photo-Activity using the Fotoscope?* | n=18 |  |
| Not useful | 11.1% (2) | n/a |
| Useful | 77.8% (14) |  |
| Very Useful | 11.1% (2) |  |
| Missing | 0 |  |
| *Specifically:* |  |  |
| *The interview with the person's loved one with dementia about his/her interests and preferences using the questions in the Fotoscope?* | n=18 |  |
| Not useful | 16.7% (3) | n/a |
| Useful | 55.6% (10) |  |
| Very Useful | 27.8% (5) |  |
| Missing | 0 |  |
| *Creating a personal profile with interest themes* | n=18 |  |
| Not useful | 22.2% (4) | n/a |
| Useful | 44.4% (8) |  |
| Very Useful | 33.3% (6) |  |
| Missing |  |  |
| *While preparing, did you also visit the general Themes page?* | n=18 |  |
| Yes | 83.3% (15) | n/a |
| No | 0 |  |
| Missing | 16.7% (3) |  |
| *While preparing, did you visit the User Guide page?* | n=18 | n/a |
| Yes | 61.1% (11) |  |
| No | 33.3% (6) |  |
| Missing | 5.6% (1) |  |
| *While preparing, or after preparing, did you also visit the Information page?* | n=18 |  |
| Yes | 50.0% (9) | n/a |
| No | 44.4% (8) |  |
| Missing | 5.6% (1) |  |
| *Did you use the Favorites page?* | n=18 |  |
| Yes | 77.8% (14) | n/a |
| No | 22.2% (4) |  |
| Missing | 0 |  |
| *What did you think of the Favorites page?* | n=18 |  |
| Not useful | 22.2% (4) | n/a |
| Useful | 50.0% (9) |  |
| Very Useful | 22.2% (4) |  |
| Missing | 5.6% (1) |  |
| *Did you use the Joker page?* | n=18 |  |
| Yes | 38.9% (7) | n/a |
| No | 61.1% (11) |  |
| Missing | 0 |  |
| *What did you think of the Joker page?* | n=18 |  |
| Not useful | 16.7% (3) | n/a |
| Useful | 44.4% (8) |  |
| Very Useful | 11.1% (2) |  |
| Missing | 27.8% (5) |  |
| *What grade would you give the Fotoscope as a tool to have a conversation with the person with dementia?* | n=23 |  |
| M (SD) | 7.7 (1.3) | n/a |
| *What grade do you give the Photo-Activity/ General Conversation Activity, as an enjoyable activity for people with dementia in the nursing home? (0 = not at all enjoyable; 10= very enjoyable)* | n=18 | n=24 |
| M (SD) | 7.8 (1.5) | 7.5 (1.10) |
| *How would you rate the Photo-Activity, done through the Fotoscope app/ general Conversation activity as a means of getting to know residents with dementia in the nursing home better?* |  |  |
| *(0=you don't get to know someone at all through it; 10=you get to know someone very well through it)* | n=18 | n=24 |
| M (SD) | 7.8 (1.8) | 7.3 (1.16) |
| **Learnability** |  |  |
| *What was your experience in learning to use the Fotoscope?* | n=18 |  |
| Difficult | 5.6% (1) | n/a |
| A little difficult | 16.7% (3) |  |
| Easy | 66.7% (12) |  |
| Very Easy | 11.1% (2) |  |
| Missing |  |  |
| *What was your experience in capturing the interests of the person with dementia using the interest questionnaire on the Fotoscope profile page?* | n=18 |  |
| Difficult | 0 | n/a |
| A little difficult | 5.6% (1) |  |
| Easy | 77.8% (14) |  |
| Very Easy | 16.7% (3) |  |
| Missing |  |  |
| *What was your experience of creating category buttons using the Theme Picker?* | n=18 |  |
| Difficult | 0 | n/a |
| A little difficult | 16.7% (3) |  |
| Easy | 66.7% (12) |  |
| Very Easy | 16.7% (3) |  |
| Missing | 0 |  |
| *What was your experience in selecting photos and placing them in the Conversation Basket?* | n=18 |  |
| Difficult | 0 | n/a |
| A little difficult | 5.6% (1) |  |
| Easy | 72.2% (13) |  |
| Very Easy | 22.2% (4) |  |
| Missing | 0 |  |
| *What was your experience in labelling photos as Favourites on the Favourites page?* | n=18 |  |
| Difficult | 5.6% (1) | n/a |
| A little difficult | 0 |  |
| Easy | 77.8% (14) |  |
| Very Easy | 16.7% (3) |  |
| Missing | 0 |  |
| *What was your experience in using the Joker page?* | n=18 |  |
| Difficult | 0 | n/a |
| A little difficult | 5.6% (1) |  |
| Easy | 50.0% (9) |  |
| Very Easy | 16.7% (3) |  |
| Missing | 27.8% (5) |  |
| **Adoption** |  |  |
| *Do you think the Fotoscope app can be a part of daily (care) activities in the nursing home?* | n=18 |  |
| Yes | 77.8% (14) | n/a |
| No | 22.2% (4) |  |
| Missing | 0 |  |
