# Supplementary material for: A Digital Photo Activity Intervention for Nursing Home Residents With Dementia and Their Carers: Mixed Methods Process Evaluation
Source: JMIR Form Res. 2025 Apr 16;9:e56586. doi: 10.2196/56586 (PMC12044310; doi:10.2196/56586)
Supplement: Multimedia Appendix 3 [file formative_v9i1e56586_app3.docx]

Supplementary File 3. Thematic analyses themes, sub-themes and sample data extracts

| **Main Theme: Context** |  |  | **Sample Data Extract (Experimental group)** |  | **Sample Data Extract (Control group)** |
| --- | --- | --- | --- | --- | --- |
| Sub-themes | Sub-sub themes |  |  |  |  |
| Time |  | - | “Planning the Photo-Activity is very important because of our workload. Time and sufficient staff presence are very essential to consider.” (Formal Carer #109, Photo-Activity) | - | “I found it very time-consuming. I felt very much the pressure because it was a lot in a short period of time.” (Formal Carer #218, control activity) |
|  |  | - | I applied the activity each time before or after my shift due to staff occupancy.” (Formal Carer #205, Photo-Activity) |  |  |
| Materials |  | - | "...arrange laptop and gather tablet.” (Formal Carer #117) | - | None |
|  |  | - | Had brought my iPad from home” (Formal Carer #203) |  |  |
| Logistics |  | + | “(did the Photo-Activity) in Madam’s own studio, in her own environment” (Carer #201) | - | Finding a quiet place in a nursing home is difficult.” (Formal Carer #003) |
| **Main Theme: Implementation** |  |  | **Sample Data Extract (Experimental group)** |  | **Sample Data Extract (Control group)** |
| Sub-themes | Sub-sub themes |  |  |  |  |
| Duration | Duration of online training | - | Keep it short and clear. Don’t talk too much around it.” (Formal Carer #230) | - | “Introduction slightly shorter and show conversation as an example” (Formal Carer #108) |
|  |  | - | “Too long, lots of repetition” (Formal Carer#119) |  |  |
|  | Duration of activity | + | "We were both chatterboxes and if I didn’t set an alarm clock we could go on for an hour with the Photo-Activity” (Formal Carer #201) | + | "We managed to have a pleasant conversation within this time. Longer could have been too, but at least it was enough time.” (Formal Carer #102) |
|  |  | + | “Liked it very much, had more to say” (Formal Carer #205) | + | "Madam can tell a lot and can keep it up for a very long time.” (Formal Carer #103) |
|  |  | - | “Concentration (of the resident) decreases after 20 minutes” (Formal Carer #001, resident #001,GDS 5) | - | "15 minutes is long enough, people get restless and you run out of conversation material. ” (Formal Carer #116,) |
|  |  | - | “For her (the resident), 30 minutes was a bit too long.” (Formal Carer #105, resident #121, GDS 5 ) | - | "Usually 20 minutes max” (Formal Carer #010) |
| Support and Resources |  | + | Always available” (Formal Carer #115) | + | Have appreciated J.T.’s (researcher) personal approach” (Formal Carer #111) |
|  |  | + | I found it nice to call if I couldn’t find something clear or knew what to do. J.T. (researcher) was able to help me well with her explanation, I understood what to do.” (Formal Carer #201) | + | Sheets…give clear information” (Formal Carer #118) |
|  |  | +/- | …expand manual with explanation on how to easily download it onto something” (on tips for improvement, Formal Carer #119) | +/- | “…a video on how the conversation between employee and resident should go” (on tips for improvement, Formal Carer #108) |
| Communication between Formal/Informal Carer |  | - | Would really have liked to have been more involved with what was happening in the nursing home as I wanted to see if it improved my mother’s mood.” (Informal Carer #101) | - | What I missed from the care organization- what was discussed and looked at and how did mum react to it?” (Informal Carer #202) |
|  |  | - | “Heard nothing at all about it.” (Informal Carer #109 | - | “No feedback on conversations or their frequency” (Informal Carer #228) |
| Digital Aspect | Preference for in-person training/demonstration | + | You don’t have to go anywhere…can just go from your workplace” (Formal Carer #105) | +/- | Teams effective, but less open” (Formal Carer #010) |
|  |  | - | Perhaps more guidance on the computer part” (reply to how to improve the online training, Formal Carer #205) | - | “Personally, I prefer to do it 'live' (not digitally)” (Formal Carer #111) |
|  |  | - | Prefer to watch someone do it live”(reply to how to improve the online training, Carer #117) |  |  |
|  | App not for everyone | +/- | Nice app. Doesn’t connect with everyone.” (Formal Carer #117, with resident #123, GDS 5 ) |  | N/A |
|  |  | +/- | “Very good (app). Albeit not suitable for everyone. Everything digital, this is something that the target group is not familiar with” (Formal Carer #005; resident #005, GDS 6) |  |  |
| Smiley Face Assessment Scale |  | - | Miss (resident) could not understand the Smileys” (Formal Carer #224, resident 221, GDS 6) | - | She (resident) did not understand the emotions. She could no longer tell her own emotions.” (Formal Carer #234, resident 218, GDS 5) |
|  |  | - | “In some moments this was difficult because they didn’t look at the feeling at that moment, but in a very broad sense and they added everything, such as living in a nursing home.” (Formal Carer #101, resident 101, GDS 5) | - | “Miss (resident) linked it to persons and did not understand the assignment” (Formal Carer #204, resident 204, GDS 5) |
| **Main Theme: Mechanisms of Impact** | |  | **Sample Data Extract (Experimental group)** |  | **Sample Data Extract (Control group)** |
| Sub-themes | Sub-sub themes |  |  |  |  |
| Black and White Photos |  | - | The photos were black and white and therefore unclear” (Formal Carer #005) |  | N/A |
|  |  | -/+ | Sometimes I got the comment whether it couldn’t have been in colour, but they mostly liked black and white pictures too” (Formal Carer #101) |  |  |
|  |  | - | I was sorry we didn’t have coloured photos. Some things really needed colour. Even my client asked me why I didn’t have colour photos.” (Formal Carer #105 with resident #109) |  |  |
|  |  | -/+ | I would personally like it better if the pictures were in colour, but for the resident, this did not matter.” (Formal Carer #105 with resident #121) |  |  |
|  |  | - | “Colour more fun.” (Formal Carer #115) |  |  |
| Amount of Photos in Database |  | + | What I really like is that there is a lot of variety in the pictures. This gives a wide choice of conversation topics.” (Formal Carer #101) |  |  |
|  |  | - | Few photos (in) certain themes, for example, pets: dogs, cats.” (Formal Carer #001) |  |  |
|  |  | - | The number of photos available- there could be more photos on it for me. Found it limited.” (Formal Carer #230) |  |  |
|  |  | - | “Resident had wanted to see more ballet pictures but there were none.” (Formal Carer #105) |  |  |
| Conversation | Residents would like to continue | + | “(Yes)…in a while with hopefully some new pictures” (Resident #119) | +/- | “Doesn't matter much…but not in the morning.” (Resident #232) |
|  |  | + | “(Yes)…with new pictures”(Resident #131) | + | “(Yes)…When it's about the piano or other subjects I like.” (Resident #120) |
|  |  | + | “(Yes)…if the caregiver also wants this.” (Resident #211) | +/- | “I don’t know.” (Resident #106) |
|  | Carers learned more about the resident | + | “You really take time with someone because you sit down with them for half an hour. Normally, you provide the care and then you leave. As a result, the resident talks more about things you wouldn’t normally hear. I also saw her smiling more and she came to the living room more often.” (Formal Carer #105, Fotoactivity) | + | I now know a little more about her past, as far as she could still tell.” (Formal Carer #204) |
|  |  | + | “During care, of course we chat plenty but here I went a bit deeper into the past, so yes I did get to know her much better than I already did.” (Formal Carer #201, Fotoactivity) | + | “I got to know more about sir, and I can approach sir much better now” (Formal Carer #103) |
| Fotoscope | Usability | + | Great usability, easy to use.” (Formal Carer #109) |  | N/A |
|  |  | + | “Nice and simple.” (Formal Carer #005) |  |  |
|  | Design | + | Fotoscope looks very clear, it is pleasant to use.” (Formal Carer #101) |  |  |
|  |  | + | “Orange, beautiful. The app looks good.” (Formal Carer #105) |  |  |
|  | Technical Issues | - | Sometimes it was annoying that the app didn’t quite work on the tablet, and I would have to grab the laptop again.” (Formal Carer #201) |  |  |
|  |  | - | …with Favorites page, namely saved pictures were duplicated or lost.” (Formal Carer #216) |  |  |
|  |  | - | “Trouble now was I had multiple clients that I had to re-enter all the data every time.” (Formal Carer #105) |  |  |
|  | Social Interaction | + | Just 1-on-1 with a person, socialising with some sweets and drinks. Rest, not that rush or I have to do this and this. Just sit and chat and get to know each other better.” (Formal Carer #201) | + | Experienced it as pleasant, as Miss (resident) herself kept indicating she really likes one-to-one.” (Formal Carer #226) |
|  |  | + | “…it triggers more conversation, especially for the quieter ones, this can work up to a conversation.” (Formal Carer #201) | - | “Personally it has no added value for me. This is what I do daily in my work- speak to residents every day.” (Formal Carer #116) |
